# Ecological and intrinsic drivers of foraging parameters of Eurasian lynx at a continental scale

**DOI:** 10.1111/1365-2656.14228

**Published:** 2024-11-26

**Authors:** Teresa Oliveira, Jenny Mattisson, Kristina Vogt, John Linnell, John Odden, Julian Oeser, Joseph Premier, Mariano Rodríguez‐Recio, Elisa Belotti, Ludek Bufka, Rok Černe, Martin Duľa, Urša Fležar, Andrej Gonev, Micha Herdtfelder, Marco Heurich, Lan Hočevar, Tilen Hvala, Tomáš Iľko, Raido Kont, Petr Koubek, Jarmila Krojerová‐Prokešová, Jakub Kubala, Marko Kübarsepp, Josip Kusak, Miroslav Kutal, Beňadik Machciník, Peep Männil, Dime Melovski, Paolo Molinari, Aivars Ornicāns, Aleksandar Pavlov, Maruša Prostor, Vedran Slijepčević, Peter Smolko, Branislav Tam, Miha Krofel

**Affiliations:** ^1^ Biotechnical Faculty University of Ljubljana Ljubljana Slovenia; ^2^ Norwegian Institute for Nature Research Trondheim Norway; ^3^ Foundation KORA (Carnivore Ecology & Wildlife Management) Ittigen Switzerland; ^4^ Department of Forestry and Wildlife Management Inland Norway University of Applied Sciences Koppang Norway; ^5^ Norwegian Institute for Nature Research Oslo Norway; ^6^ Geography Department Humboldt‐Universität zu Berlin Berlin Germany; ^7^ Department of National Park Monitoring and Animal Management Bavarian Forest National Park Grafenau Germany; ^8^ Department of Biodiversity, Ecology and Evolution. Faculty of Biological Sciences Complutense University of Madrid Madrid Spain; ^9^ Faculty of Forestry and Wood Sciences Czech University of Life Sciences Prague Prague Czech Republic; ^10^ Department of Research and Nature Protection Šumava National Park Administration Kašperské Hory Czech Republic; ^11^ Slovenia Forest Service Ljubljana Slovenia; ^12^ Department of Forest Ecology, Faculty of Forestry and Wood Technology Mendel University in Brno Brno Czech Republic; ^13^ Carnivore Conservation Programme Friends of the Earth Czech Republic Olomouc Czech Republic; ^14^ Macedonian Ecological Society Skopje North Macedonia; ^15^ Forest Research Institute Baden‐Wuerttemberg Breisgau Germany; ^16^ Chair of Wildlife Ecology and Management, Faculty of Environment and Natural Resources University of Freiburg Freiburg Germany; ^17^ Administration of the Muránska Planina National Park with Headquarters in Revúca Revúca Slovakia; ^18^ University of Tartu Tartu Estonia; ^19^ Institute of Vertebrate Biology of the Czech Academy of Sciences Brno Czech Republic; ^20^ Department of Zoology, Fisheries, Hydrobiology and Apiculture, Faculty of AgriSciences Mendel University in Brno Brno Czech Republic; ^21^ Faculty of Forestry, Department of Applied Zoology and Wildlife Management Technical University in Zvolen Zvolen Slovakia; ^22^ DIANA—Carpathian Wildlife Research Banská Bystrica Slovakia; ^23^ Estonian Environment Agency Tartu Estonia; ^24^ Veterinary Faculty University of Zagreb Zagreb Croatia; ^25^ College of Sciences Koc University Istanbul Turkey; ^26^ State Nature Conservancy of Slovak Republic Landscape Area Strážov Mountains Administration Považská Bystrica Slovakia; ^27^ Italian Lynx Project Tarvisio Italy; ^28^ Department of Hunting and Wildlife Management Latvian State Forest Research Institute “Silava” Salaspils Latvia; ^29^ Karlovac University of Applied Sciences Karlovac Croatia; ^30^ Zoological Department National Zoological Garden Bojnice Bojnice Slovakia; ^31^ Institute of Animal Husbandry, Faculty of Agrobiology and Food Resources Slovak University of Agriculture Nitra Slovakia

**Keywords:** Eurasian lynx, Europe, foraging, handling time, human impact, inter‐kill interval, prey availability, scavengers

## Abstract

1. The estimation of foraging parameters is fundamental for understanding predator ecology. Predation and feeding can vary with multiple factors, such as prey availability, presence of kleptoparasites and human disturbance. However, our knowledge is mostly limited to local scales, which prevents studying effects of environmental factors across larger ecological gradients.
2. Here, we compared inter‐kill intervals and handling times of Eurasian lynx (*Lynx lynx*) across a large latitudinal gradient, from subarctic to the Mediterranean ecosystems, using a standardised dataset of predicted adult ungulate kills from 107 GPS‐collared lynx from nine distinct populations in Europe. We analysed variations in these two foraging parameters in relation to proxies reflecting prey availability, scavengers' presence and human disturbance, to improve our understanding of lynx predation at a continental scale.
3. We found that inter‐kill intervals and handling times varied between populations, social status and in different seasons within the year. We observed marked differences in inter‐kill intervals between populations, which do not appear to be driven by variation in handling time. Increases in habitat productivity (expressed by NDVI, used as a proxy for prey availability) resulted in reduced inter‐kill intervals (i.e. higher kill rates).
4. We observed less variation in handling (i.e. feeding) times, although presence of dominant scavengers (wild boars and brown bears) and higher human impact led to significantly shorter handling times. This suggests that kleptoparasitism and human disturbance may limit the energetic input that lynx can obtain from their prey. We also observed that the human impact on foraging parameters can be consistent between some populations but context‐dependent for others, suggesting local adaptations by lynx.
5. Our study highlights the value of large‐scale studies based on standardised datasets, which can aid the implementation of effective management measures, as patterns observed in one area might not be necessarily transferable to other regions. Our results also indicate the high degree of adaptability of these solitary felids, which enables them to meet their energy requirements and persist across a wide range of environmental conditions despite the constraints imposed by humans, dominant scavengers and variable prey availability.

## Graphical abstract

Our paper is the first study showing the variation of standardised foraging parameters of a large carnivore across large environmental gradients at a continental scale. We highlight the often‐neglected human impact on foraging abilities. We also documented an exceptionally high degree of adaptability of solitary felids.

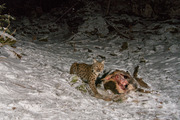

## 1 INTRODUCTION

Predation is an essential ecological process where generally a predator searches for prey, captures and consumes it. The estimation of inter‐kill intervals is fundamental to understanding predation and foraging patterns in terrestrial predators. The inter‐kill interval is composed of two parameters: searching time (the time spent searching for prey) and handling time (capturing and consuming prey) (Holling, 1959), which are indicative of energy expenditure and acquisition by a predator, thus directly influencing its survival. Furthermore, analysing inter‐kill intervals allows assessing how predation affects prey population dynamics, particularly when estimates of these intervals consider the type of prey and time of the year (Metz et al., 2012; Vucetich et al., 2011).

Foraging parameters (i.e. inter‐kill interval, searching and handling times) can vary according to multiple factors, such as availability, seasonality and distribution of prey (Elbroch et al., 2016), density of predators, presence of competitors and kleptoparasites (Smith et al., 2015; Zimmermann et al., 2015) and individual variability (Pettorelli et al., 2015). Understanding the impact of these factors can reveal how predators handle different environmental conditions to obtain the necessary energy input (Merrill et al., 2010; Sunquist & Sunquist, 1989). Since many solitary large predators tend to predate on large prey (similar size or larger than themselves, although prey size selection can vary greatly; see Bates‐Mundell et al., 2024) and thus feed on a carcass for several days, kleptoparasitism by scavengers can also importantly affect the predator's feeding behaviour and kill rates (i.e. number of kills per unit of time) (Allen et al., 2021; Krofel et al., 2012). Moreover, these factors seldom act independently, and their intertwined nature makes disentangling the influence of individual variables on variation in foraging parameters challenging.

Because of the considerable effort and resources required to obtain large datasets on the foraging parameters of large carnivores, foraging studies are often conducted with relatively small sample sizes. Therefore, our understanding of these parameters is typically limited to local scales (i.e. single study sites). As such, the effect of environmental factors on the foraging behaviour of predators across extensive ecological gradients is less understood. Compiling data from several populations increases sample sizes and allows for an assessment of predators' foraging behaviour across large‐scale ecological gradients. This could deepen biological insights into how variables are connected and interact with others (confounding factors) as well as potential global patterns and local or context‐dependent variation (Mumme et al., 2023; Oeser et al., 2023). To our knowledge, so far only one study has adopted this large‐scale approach (Cristescu et al., 2022). This study revealed significant variation in the ungulate kill rates of mountain lions (*Puma concolor*), but the authors acknowledged the lack of data standardisation due to different methodologies used to estimate foraging parameters in different populations, which limited the comparability of their datasets.

Europe contains diverse habitats and environmental conditions, with large gradients of human presence and differences in prey availability, as well as increasing populations of large carnivores. This makes it suitable for exploring ecological adaptations of large predators under different environmental conditions. The Eurasian lynx (*Lynx lynx*) is a large solitary felid of conservation concern, whose main prey in Europe are wild ungulates, especially the European roe deer (*Capreolus capreolus*) (Khorozyan & Heurich, 2023). Small prey (e.g. hares, rodents and birds) can also play an important role in lynx diet in some populations (Krofel et al., 2011; Melovski et al., 2022; Vogt et al., 2018), as well as domestic and semi‐domestic prey in Scandinavia (Mattisson, Odden, et al., 2011; Odden et al., 2006). These differences in prey availability and diversity could drive variation in lynx foraging parameters across Europe. Several studies have explored variation in kill rates at a local scale in relation to ecological factors, such as human impact, environmental productivity or presence of heterospecifics (e.g. Krofel & Jerina, 2016; Nilsen et al., 2009; Okarma et al., 1997). However, these studies have limited variation of such factors, due to small datasets and restricted spatial scale, which hampers our understanding of the real impact of these factors on kill rates. Additionally, because foraging parameters are often estimated differently among research groups, the results are not directly comparable between studies.

**FIGURE 1:**
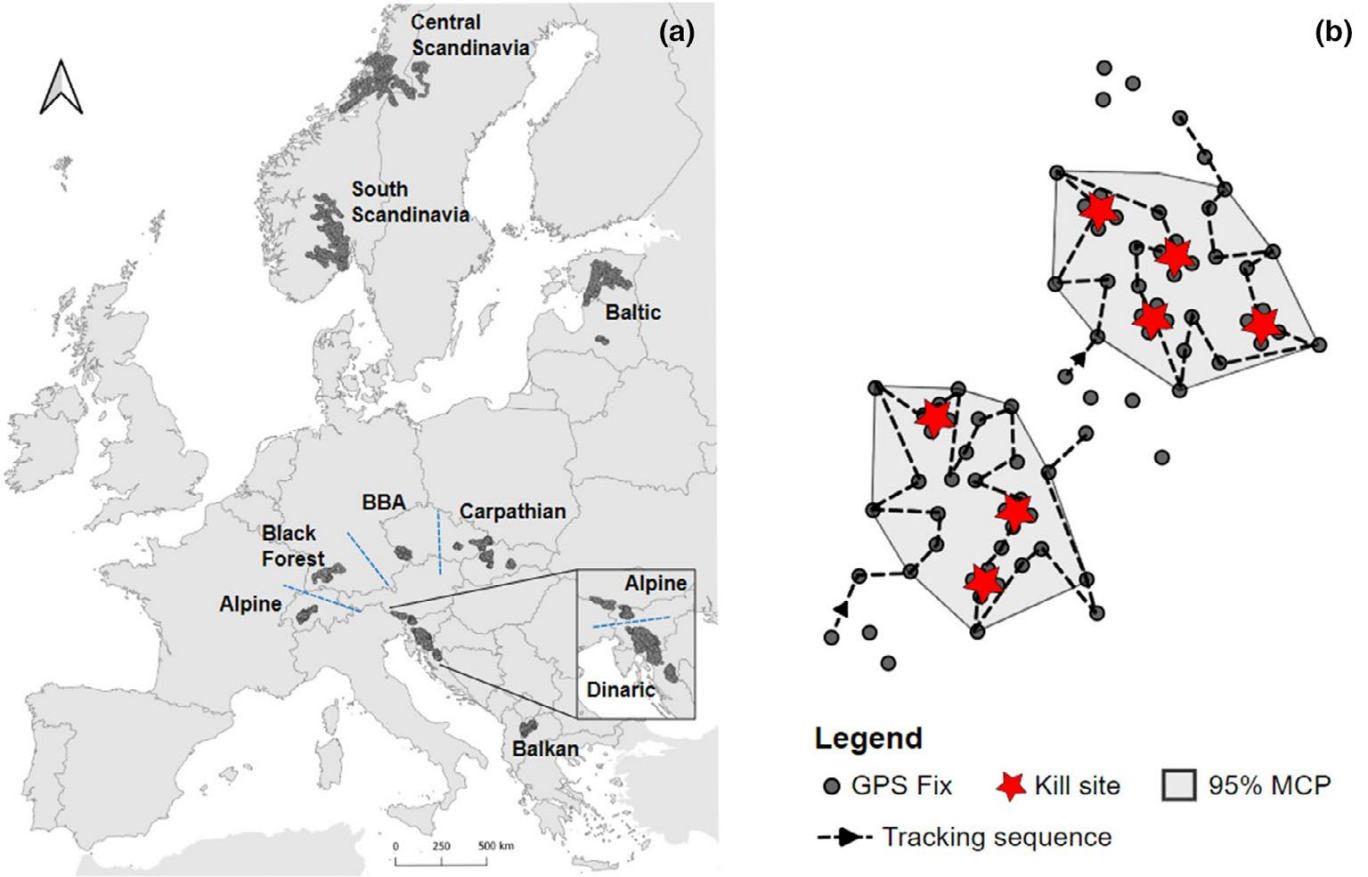
(a) Spatial distribution of lynx GPS data within each population. BBA stands for Bohemian‐Bavarian‐Austrian population. (b) Representation of tracking sequences. The polygons represent the 95% MCP of the GPS data within the tracking sequences. Inter‐kill intervals and handling times were estimated within tracking sequences.

Here, we evaluated variation in foraging parameters of Eurasian lynx at a continental scale in relation to several ecological drivers using a standardised dataset of GPS location clusters (hereafter, clusters) with predicted adult ungulate kills from nine lynx populations across Europe (Figure 1a). We focused on the relative changes in two foraging parameters related to kill rates (inter‐kill interval and handling time) of ungulates (i.e. wild, domestic and semi‐domestic ungulates >7 kg; Oliveira et al., 2022). Based on previous local studies, we identified three main ecological factors affecting foraging parameters (prey availability, human impact and presence of dominant scavengers), and we investigated their effect on the two foraging parameters at a continental scale. Furthermore, taking advantage of the large dataset available and the associated individual variability, we explored how these two foraging parameters, varied between populations and with social status (individually and as a function of the ecological drivers), as well as along the year. Our study provides a comprehensive, large‐scale understanding of lynx foraging parameters, which is relevant not only for lynx conservation, but also for wildlife management in human‐dominated landscapes.

## 2 METHODS

### 2.1 Data available

We obtained GPS data from nine lynx populations across Europe (Figure 1a) and selected data periods (hereafter, tracking sequences; Figure 1b) of a minimum of 21 consecutive days, with at least three scheduled GPS fixes per night (between 5 pm and 7 am), to ensure a reliable estimation of inter‐kill intervals and handling times (Nilsen et al., 2009; Oliveira et al., 2022). We selected a minimum of three fixes per night because this allows the detection of 95% of the clusters reflecting adult wild ungulate kill sites (Oliveira et al., 2022). Because unsuccessful GPS fix attempts can result in lower sampling rates than scheduled, we allowed a tracking sequence to have a maximum of two consecutive days with two fixes/night or 1 day with one fix/night only. For each tracking sequence, we created a 95% MCP from the GPS data within the tracking sequence, to extract the environmental covariates (section 2.4.2; Figure 1b).

After filtering, we obtained a dataset that comprised a total of 308 tracking sequences (Table S1; Figure S1), which included 140,448 GPS fixes obtained within a total of 25,379 days from 122 lynx individuals (86 males/36 females). These data were used to generate and classify clusters (Section 2.2). The information regarding lynx capture and permit numbers are provided in Appendix S1.

### 2.2 Predicting ungulate kill sites

We built on the approach developed by Oliveira et al. (2022) to obtain clusters and created a predictive model for the studied lynx populations (Appendix S2). First, we generated clusters with a spatial buffer of 200 m, 2 days of spatial window and a minimum number of two fixes. Secondly, we used the random forest algorithm and several cluster characteristics to build a binary predictive model able to distinguish between clusters reflecting adult ungulate kills (1) from non‐kills and small prey (0) (i.e. non‐ungulate prey and small ungulates with body mass <7 kg, including neonatal or juvenile ungulates, Table S1; for further details see Oliveira et al., 2022). We used this approach because our sample size for non‐kills and small prey was overall smaller than the adult ungulate kills. We built the binary predictive model with available data on field‐checked clusters (i.e. clusters visited in the field and searched for prey remains), using a total of 3434 clusters visited in the field (1312 clusters considered as non‐kills, 440 connected with confirmed small prey, and 1682 connected with confirmed ungulate kills). We obtained a model accuracy of 85.5% (CI 95%: 83.0–87.8), with sensitivity and specificity of 86.2% and 84.7%, respectively (Appendix S2).

### 2.3 Foraging parameters

We estimated inter‐kill intervals and handling (i.e. feeding) times within each tracking sequence. The inter‐kill interval was estimated as the time elapsed between the start of a kill *k*_*n*_ (considered as the first GPS fix of the cluster) and the start of the next kill *k*_*n*+1_. Inter‐kill interval provides similar information as kill rates, which reflects the number of prey killed per unit of time. Shorter inter‐kill intervals indicate higher kill rates and vice versa. Handling time was estimated as the time between the start of *k*_*n*_ and its abandonment (first and last GPS fixes of the given cluster, respectively). We did not include searching time (i.e. time elapsed between abandoning kill *k*_*n*_ and the start of kill *k*_*n*+1_) in our analyses because this parameter was highly correlated with inter‐kill intervals (Pearson's correlation *r* = 0.95).

### 2.4 Predictor covariates

#### 2.4.1 Individual factors

We classified the age of lynx individuals (males and females) as adults (2 years or older) or sub‐adults (between 1 and 2 years old). We considered mothers with dependent kittens from the end of the denning period (defined as 2 months from birth; Krofel et al., 2013) up to the date when the kittens dispersed as family groups. The denning period was excluded from the analyses. This was because the predictive model did not perform well during this period when females spend considerable time at the den and effectively become central‐place foragers. We used available field data (e.g. camera trapping, snow tracking) for each litter to detect the approximate time of break‐up of a family group, that is when kittens no longer accompany the female. Data from sub‐adult lynx (both males and females) was considered while building the predictive model (Section 2.2), but the output of the predictive model for this social status was not included in further analyses (i.e. variation of foraging parameters). The reason for removing this category was connected to the difficulties in reliably separating and classifying, clusters between small prey (or non‐kills) and adult ungulate kills for sub‐adult lynx, as they tend to stay for long periods even at small prey and may engage more in scavenging behaviour than adults (Krofel et al., 2011).

#### 2.4.2 Environmental factors

For each environmental covariate, we extracted values at the kill site and within the tracking sequence (95% MCP, Figure 1b; Table S3). The values extracted at the kill site location were solely used for the models using handling time as the response variable, while the values extracted within tracking sequences were considered for the models using inter‐kill interval as the response variable, except for some proxies of scavengers' presence (see Table S3 for descriptions, units, data resolution and extraction procedures for each covariate).

Because we lacked reliable and comparable data on prey density across all populations, we considered three covariates as proxies for prey availability: lynx home‐range size, estimated with all GPS data available for a given individual (km^2^, 85% MCP), forest edge density (Ruiz‐Villar et al., 2023) and the Normalised Difference Vegetation Index (NDVI, 250 m × 250 m; Didan, 2021). We included home‐range size as a proxy because it is often connected with prey availability, as larger home‐ranges generally reflect lower prey availability, although home‐range size can also vary with other factors, such as lynx density and female presence (Aronsson et al., 2016; Molinari‐Jobin et al., 2007; Schmidt, 2008). We defined home range as the MCP at 85% to exclude excursions outside the areas used more frequently. We included forest edge density because it reflects varying levels of landscape heterogeneity and configuration, and it is linked to the presence and abundance of the lynx's primary prey species in Europe (i.e. the roe deer; Morellet et al., 2011). High forest edge density values correspond to a higher density of forest edges and, therefore, a more suitable habitat for roe deer. Additionally, we included NDVI as an indicator of plant productivity previously used as a proxy for roe deer density (e.g. Melis et al., 2010). The values per population are available in Figure S3.

We considered large scavenger occurrence data (presence/absence) collected at the European level (brown bear *Ursus arctos* and wolverine *Gulo gulo*, Kaczensky et al., 2021; wild boar *Sus scrofa*, Linnell et al., 2020) as indicators for a scavengers' species presence in the area used by lynx (Table S3). We included covariates reflecting scavengers' presence because these scavengers are known to affect lynx handling times and kill rates through kleptoparasitism (Duľa & Krofel, 2020; Krofel et al., 2012; Mattisson, Andren, et al., 2011). We used two measures to account for scavengers' presence: the number of scavenger species potentially present at the location of the kill according to occurrence maps (i.e. general presence in the area, we did not have information whether the scavenger was actually present at a given kill) and scavenger presence within each tracking sequence (i.e. the proportion of the area of each tracking sequence overlapping with scavenger's occurrence map), as an indication of encounter risk (Table S3; see Figure S6 for values per population).

We considered two proxies for human impact: human modification index (HMI; Theobald et al., 2020) and distance to settlements (Ferri et al., 2016) (Table S3, and see Figures S4 and S5 for values per population). Human impact is known to affect lynx habitat use in Europe (Ripari et al., 2022) and can also cause large carnivores, including lynx, to abandon their prey (Krofel et al., 2008; Smith et al., 2017). The human modification index incorporates data from human activities that alter natural systems (Theobald et al., 2020), and high values of this index indicate higher human impact. Distance to settlements reflects the Euclidean distance to the closest human settlement/build‐up area.

### 2.5 Modelling approach

We built generalised additive models (GAMs; Wood, 2017) to test our hypotheses. We considered inter‐kill interval and handling time as response variables and used the Gamma error distribution family with a log link. We first built a series of models (‘baseline models’) to check for the suitability of two random intercepts, and the effect of social status and population ID alone on foraging parameters. We included lynx ID as a random intercept (“re” basis) to account for individual variation, and the number of daily fixes used per tracking sequence, to account for potential bias due to differences in GPS fix rates among and within the study areas and individuals. We also included a seasonal covariate (month) to evaluate how inter‐kill intervals and handling times vary over the year. We specified the basis type of “month” as a cyclic cubic spline (“cc”), as there should be no discontinuity between December and January (Wood, 2017). In preliminary analyses, we also included the number of days per tracking sequence as a random intercept but, since it did not improve model performance, we removed it from further analyses. When looking at the effects of the environmental covariates, we retained the individual and population‐related covariates (i.e. population ID and social status) that significantly improved the model fit of the baseline models. Additionally, we built two more models for each environmental covariate accounting for potential group‐specific deviations of population ID and social status from the global function by including a factor‐smooth interaction (“fs” basis; Pedersen et al., 2019). If a given environmental variable showed a low degree of non‐linearity (effective degrees of freedom: EFD ~1), we tested whether including the variable as linear (i.e. without the smooth term) would improve model fit.

We compared AIC values (Akaike's information criteria; Anderson & Burnham, 2002) and calculated the difference in AIC (ΔAIC) of each model to a null model, and we then compared the ΔAIC values of models including an environmental variable to the baseline model, to evaluate the gain of adding these variables. We considered the coefficients from the model(s) with the highest support according to AIC (ΔAIC ≤ 2, Anderson & Burnham, 2002) to evaluate our hypotheses. If two models built with the same environmental covariate, with and without the factor‐smooth interaction, had a ΔAIC ≤ 2, we considered the simplest model (i.e. without the factor‐smooth interaction). We tested for collinearity between variables using a variance inflation factor (VIF) and excluded variables from the model formulation that had a factor value >3 (Zuur et al., 2010). Because the data on scavengers' presence was correlated with the population ID (VIF > 3), we excluded the latter covariate when testing the hypothesis referring to the effects of scavenger's presence/absence. All analyses and data visualisation were performed using R Statistical Software (v 4.1.2; R Core Team, 2021). Specifically, we used the *mgcv* v1.8‐42 (Wood, 2017) package to build GAMs and the *DHARMa* v0.4.6 (Hartig, 2022) and *gratia* v0.8.1 (Simpson, 2023) R packages for model diagnosis. We used the package *marginaleffects* v0.13.0 (Arel‐Bundock, 2023) to plot the output of our models as conditional predictions. For all plots showing conditional predictions, the reference category for social status is adult male, and for population ID is the Carpathian population.

## 3 RESULTS

We included 4424 predicted adult ungulate kills from 107 adult lynx (74 males/33 females) for evaluating the variation in inter‐kill intervals and handling times. Of those predicted kills, 3192 were from adult males, 718 from adult females and 514 from family groups.

### 3.1 Population, social status and seasonal variations

**FIGURE 2:**
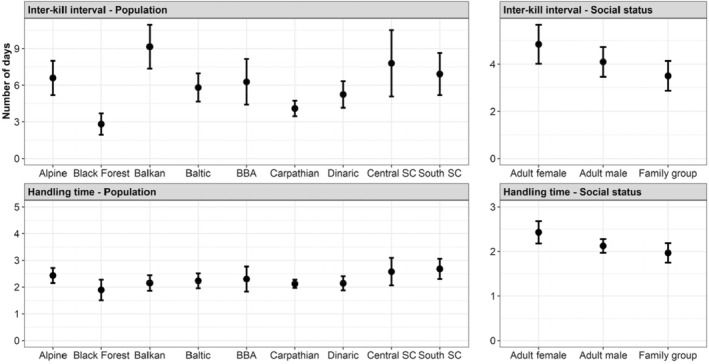
Predicted values for inter‐kill interval and handling time for the model including population ID and social status as fixed factors. SC stands for Scandinavia, and BBA for the Bohemian‐Bavarian‐Austrian population. See Figure S8 for the coefficient values per population/social status.

Inter‐kill interval and handling time varied between populations and with social status (Figure 2). Specifically, family groups showed shorter inter‐kill intervals (higher kill rates) and shorter handling times, followed by adult males and then by adult single females. We observed a higher variation of inter‐kill intervals between populations (ranging from approximately 3–9 days in the Black Forest and Balkan populations, respectively) compared to the variation of handling times (approximately 2–3 days for all populations; Figure 2).

**FIGURE 3:**
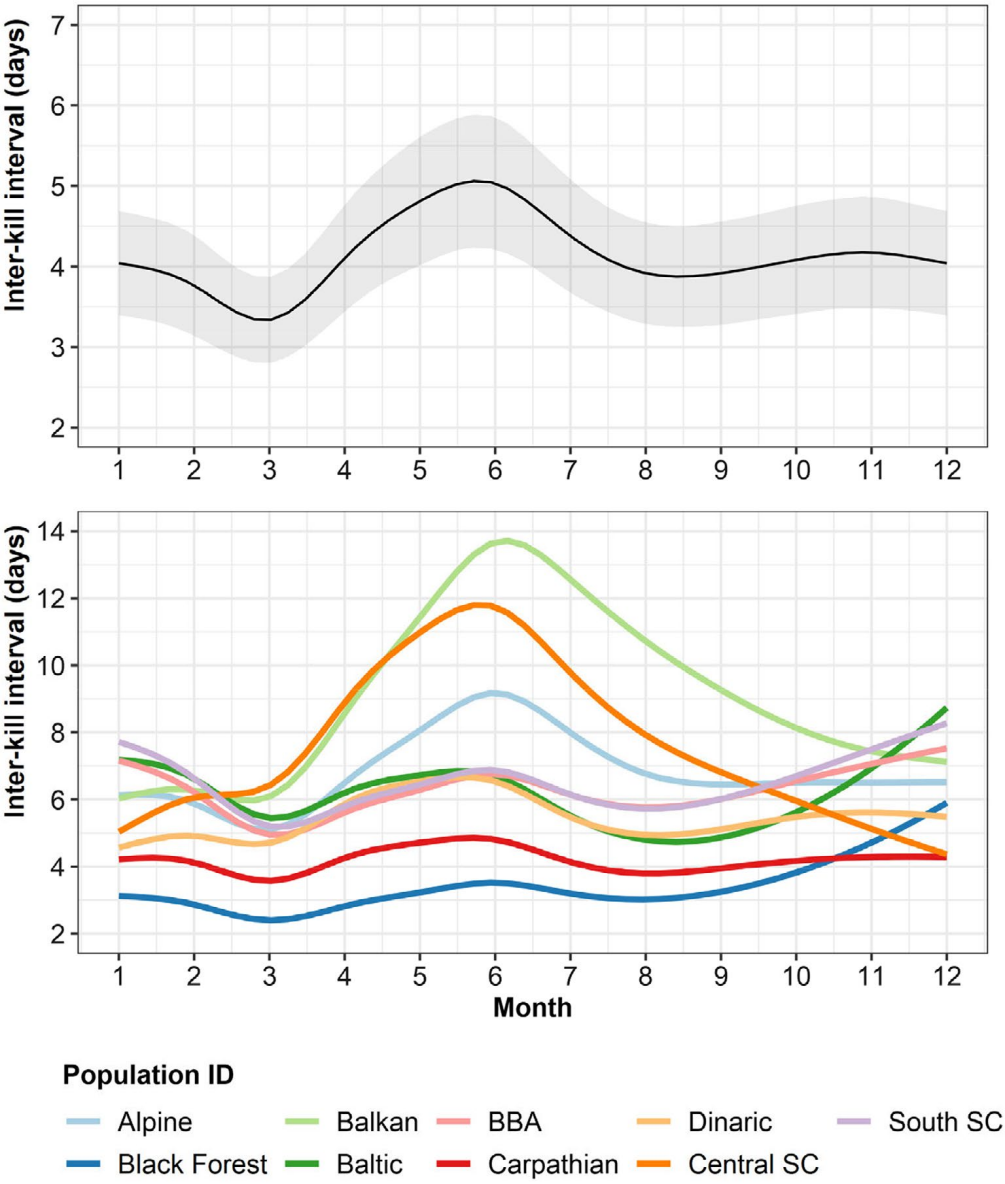
Variation in the predicted values for inter‐kill interval over the year (first panel) and by population (second panel). SC stands for Scandinavia, and BBA for the Bohemian‐Bavarian‐Austrian population.

We also observed differences in the two foraging parameters during the year. Inter‐kill intervals were longer at the beginning of summer (~5 days; May–June) and shorter in March (~3.5 days; Figure 3). However, we observed marked differences when looking at each population individually, with the Balkan, the central Scandinavian and, to a lesser extent, the Alpine populations exhibiting a stronger increase in inter‐kill intervals during the summer (Figure 3). On the other hand, the Carpathian population showed similar inter‐kill intervals throughout the year (Figure 3). Handling times varied only about 0.5 days between seasons, and this was consistent between populations (Figure S9).

Inter‐kill intervals and handling time also varied seasonally with social status. Particularly, adult single females increased their inter‐kill interval by about 2 days during summer (Figure S10). When considering handling times, we observed a similar trend between males and single females, while family groups exhibited more pronounced variation in handling time (about 1 day throughout the year; Figure S9). AIC values for all models are shown in Figure S7.

### 3.2 Prey availability

**FIGURE 4:**
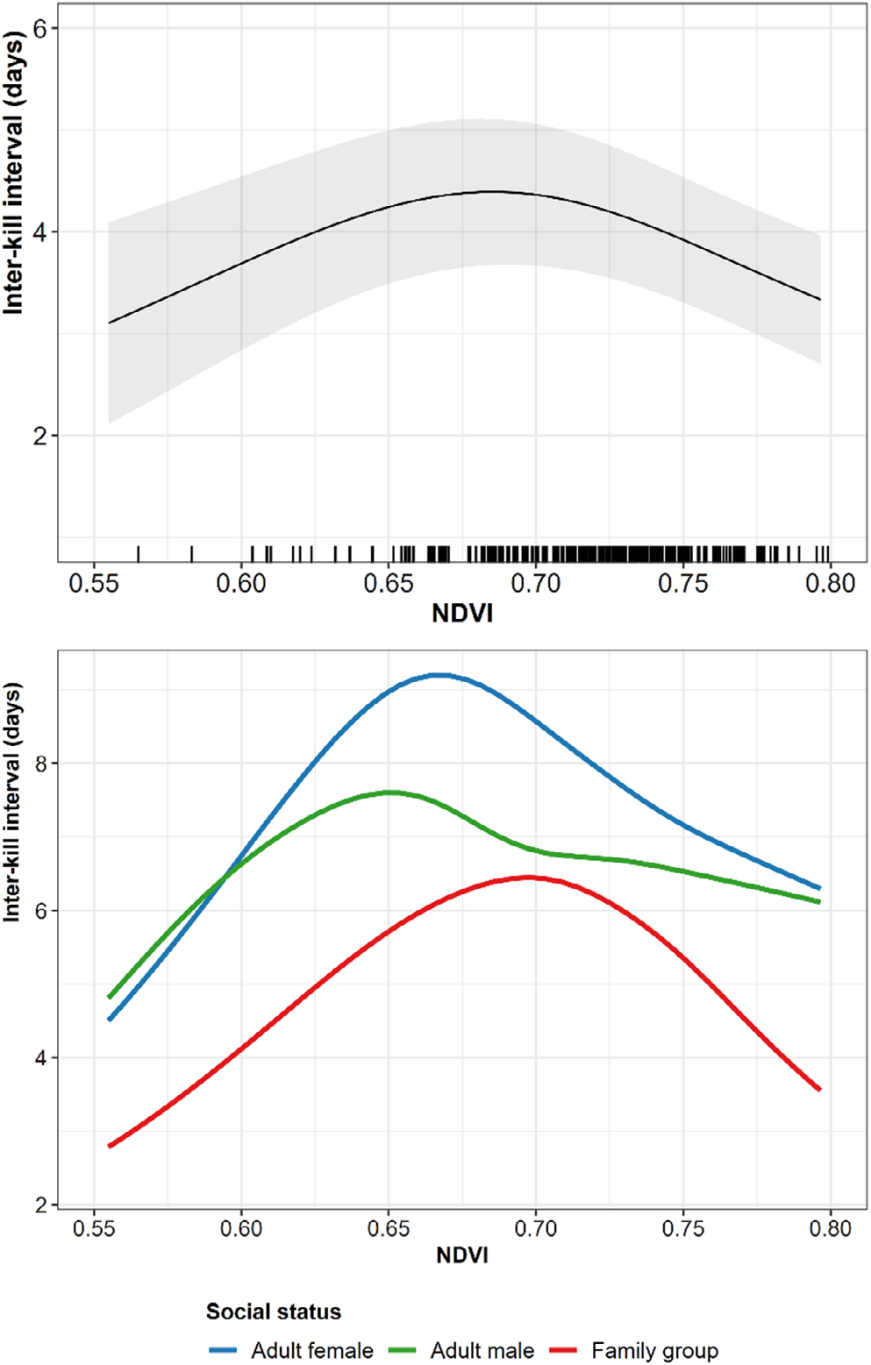
Effect of NDVI on the overall variation of inter‐kill interval (upper panel) and by social status (lower panel). The rugs on the *x*‐axis (upper panel) reflect the observed values within each tracking sequence (95% MCP, median value). The reference category for population ID is the Carpathian population.

From the covariates used as proxies for prey availability, only NDVI significantly influenced the inter‐kill interval (Figure S7). Specifically, we observed that intermediate values of NDVI (0.65–0.70) resulted in longer inter‐kill intervals, and increased values of NDVI reduced the inter‐kill interval (Figure 4). Lower NDVI values also reduced inter‐kill intervals, but there were few observations for NDVI values below 0.65 and they were restricted to a single population (Balkan; Figure S3). When decomposing the effect of NDVI by social status, we observed similarities to the general pattern, with family groups displaying the most evident decrease (>2 days), and males showing less variability in inter‐kill intervals as productivity increases (Figure 4).

### 3.3 Scavenger presence

**FIGURE 5:**
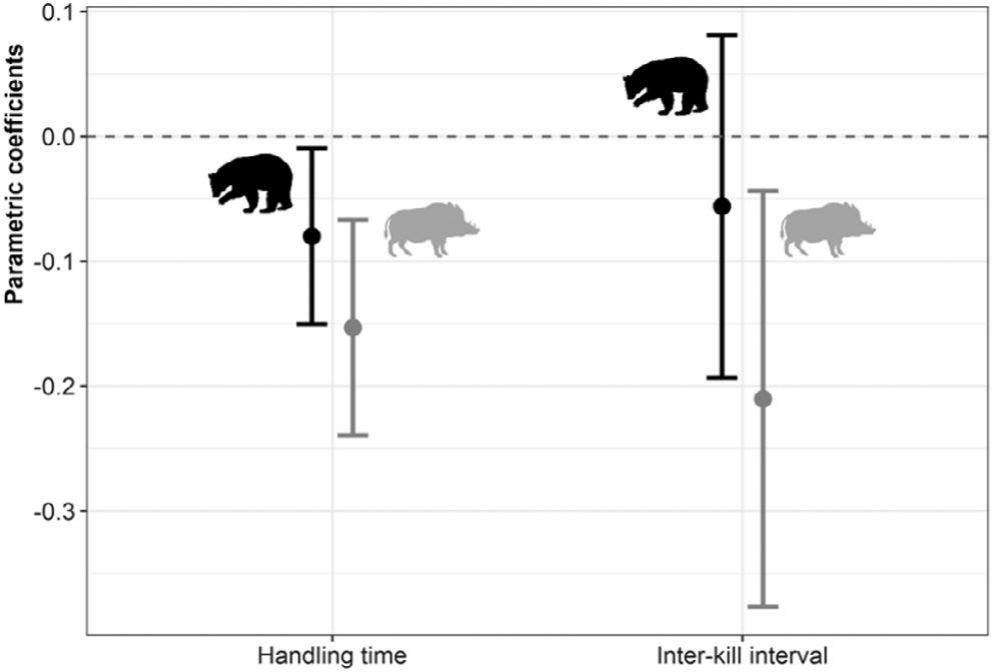
Parametric coefficients and 95% confidence intervals for the covariates reflecting the individual effect of brown bear (dark silhouette) and wild boar (grey silhouette) presence within a tracking sequence (proportion within the 95% MCP) on inter‐kill intervals and handling times. Positive coefficients indicate an increase in the foraging parameter as the presence increases, while negative values indicate a decrease in the foraging parameter. Confidence intervals crossing zero (dashed line) reflect a non‐significant trend.

We found an effect of brown bear and wild boar presence, but not of wolverine presence, on handling time and inter‐kill interval (Figure S7). Specifically, an increase in a potential encounter with a wild boar (i.e. wild boar presence within the 95% MCP of the tracking sequence) significantly decreased inter‐kill interval (higher kill rates) and shortened handling time (Figure 5). Increased presence of brown bear had a similar effect but was significant only for handling time (Figure 5). On the other hand, the model fit did not improve when considering the three large scavengers in the same model (brown bear, wild boar, and wolverine) compared to models with a single scavenger species (Figure S7). The number of scavenger species did not improve model fit for handling time (Figure S7).

### 3.4 Human impact

**FIGURE 6:**
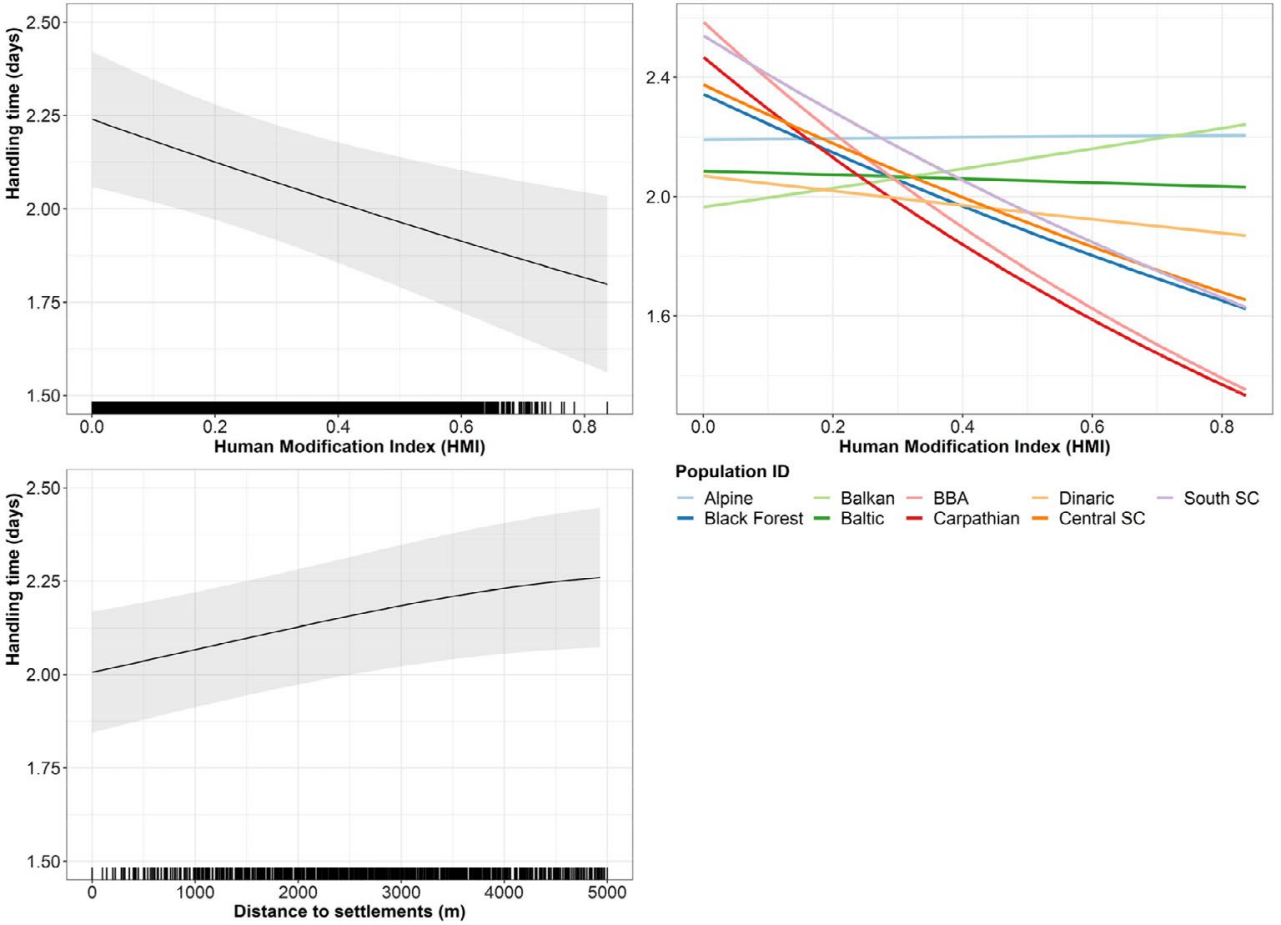
Effect of human impact on the variation of handling time per ungulate kill. The upper left panel reflects the overall impact of the human modification index on handling times, and the upper right panel reflects this impact by population. The bottom left panel reflects the overall impact of distance to settlements on handling times. The rugs on the *x*‐axis show the observed values at each kill site. SC stands for Scandinavia and BBA for the Bohemian‐Bavarian‐Austrian population.

Including human impact improved model fit for handling time but not for the inter‐kill interval (Figure S7). When looking at the effects of human impact on the variation in handling time, we observed longer handling times with increasing distance of the kill to human settlements (Figure 6). Similarly, handling time shortened by about 0.5 days as the human modification index increased from 0 to 0.8 (Figure 6). This pattern was evident for five of the nine populations. The Balkan population showed a slightly opposite trend while the Alpine, Baltic and to a lesser extent, the Dinaric populations did not seem to be affected by huma modification index (Figure 6). We also observed different patterns with social status: family groups were overall less affected by human impact, while adult males were the most affected, with handling times reduced by about 0.6 days as human modification index increased to 0.8 (Figure S11). The effect of distance to settlements on handling time was consistent between social status and populations (Figure S7).

## 4 DISCUSSION

Both foraging parameters, inter‐kill interval and handling time, varied significantly between populations and in respect to multiple ecological factors on a pan‐European scale. Our results show the high adaptability of the Eurasian lynx to a large gradient of environmental conditions, particularly to human disturbance and the presence of dominant scavengers. Such level of adaptability is not easily revealed at a local level, due to lower variation in environmental factors, or with a meta‐analysis of published data which may have low comparability. Our approach of using a standardised dataset collected across multiple populations overcomes these challenges and could be applied to other carnivore species of similar foraging ecology (e.g. several species of other felids, canids and hyenids). This could help understanding how common the level of adaptability observed in Eurasian lynx is among other large carnivores, which has implications for their conservation and wildlife management.

### 4.1 Population, social status and seasonal variations

We observed variation in lynx inter‐kill intervals between populations and throughout the year. Namely, the Balkan population showed the longest inter‐kill intervals, indicating a lower number of adult ungulates killed than in other areas. Ungulate densities are overall low within the area of distribution of the Balkan population, mostly due to past hunting management, which presents a conservation challenge (Melovski et al., 2020). Despite being an efficient predator even at low prey densities (Krofel et al., 2014; Nilsen et al., 2009), longer inter‐kill intervals suggest a higher dependence of lynx on smaller prey to obtain the necessary energy input, which was already described for populations where wild ungulate density is low (the Balkan population: Melovski et al., 2020, 2022; Scandinavia: Mattisson, Odden, et al., 2011; Odden et al., 2006). On the other hand, the Black Forest showed the shortest inter‐kill intervals, which could reflect the naïve prey present in this recent reintroduction area potentially leading to surplus killing (Duľa & Krofel, 2020), although the sample was limited to two males. Furthermore, we observed seasonal variations in inter‐kill interval, with a stronger increase in inter‐kill intervals for the Balkan population during the summer, which suggests a more pronounced switch from adult ungulates to smaller prey (including neonate ungulates) during this period (Belotti et al., 2015; Krofel et al., 2013; Mattisson et al., 2014; Nilsen et al., 2009), when compared to other populations.

The variation of handling time between the nine populations was less pronounced than the variation in inter‐kill interval. Such low variation suggests that finding adult ungulate prey (searching time) could be a limiting factor influencing inter‐kill interval. Searching time is connected to the probability of encountering prey, thus lower prey densities may lead to longer searching times (Holling, 1959). However, the time between the utilisation of adult ungulate kills can also be influenced by feeding on small prey. In this study, the search and handling time of small prey are included in the estimated search time between adult ungulate kills, as we could not account for different prey types in the estimation of foraging parameters.

The variation of foraging parameters in relation to lynx social status was consistent across populations and confirms results of previous local studies on lynx and other felids (e.g. Belotti et al., 2015; Cristescu et al., 2022; Mattisson, Odden, et al., 2011; Miller et al., 2013; Molinari‐Jobin et al., 2007; Nilsen et al., 2009; Okarma et al., 1997). Single adult females had the longest inter‐kill intervals (lowest kill rates), followed by adult males and family groups exhibited the shortest inter‐kill intervals. Females with kittens have higher nutritional demands due to raising offspring and extended periods without ungulate prey can affect reproductive success (Walton et al., 2017). Additionally, the significant differences in inter‐kill intervals between adult males and single adult females could be connected to the differences in body size and the greater movements of males, probably linked to territorial defence, which increases their energetic needs (Sunquist & Sunquist, 2002).

### 4.2 Prey availability

We considered three proxies for prey availability: NDVI, home range size and forest edge density. Among our proxies for prey availability, only plant productivity influenced inter‐kill intervals. We found that inter‐kill interval increased with increasing NDVI until peaking at intermediate values of NDVI. However, as the lower NDVI values were limited to a few points from a single population, we could not infer about its ecological meaning. Thereafter, we found that intermediate to high values of NDVI resulted in gradually shorter inter‐kill intervals, suggesting that areas with higher productivity reduce the time between utilisation of ungulate prey. However, using NDVI as an index of prey availability may represent both ungulates' density, where high NDVI values are expected to shorten inter‐kill intervals between ungulate kills and the availability of smaller prey, which is expected to increase inter‐kill intervals if the lynx switch to a higher proportion of small prey. Therefore, these two mechanisms are not easily disentangled without access to real prey densities. When looking at variation within social status, females (single females and family groups) appear to be more sensitive than males to variation in NDVI values. Such differential responses might relate to individual males roaming over more diverse habitats due to their larger home ranges and more intensive territorial marking (Krofel et al., 2017; Vogt et al., 2014). Conversely, females, particularly those accompanied by kittens, navigate primarily to secure food sources. Therefore, they might use more often, and more consistently, areas characterised by higher productivity levels.

Lynx home‐range sizes show a high variation between European populations, and we expected the inter‐kill interval to be generally shorter with smaller home range size, varying particularly by social status, as the area used by female lynx can be negatively correlated to ungulate density (Aronsson et al., 2016; Linnell et al., 2021; Schmidt, 2008; but see Molinari‐Jobin et al., 2007). Males, on the other hand, can be expected to adjust their home ranges according to female presence in the area (Sandell, 1989). However, we found no effect of home range size on inter‐kill interval. Nevertheless, the lack of trends could be attributed to local factors other than prey density (e.g. conspecific density and their sex‐ratio) which, acting independently or in combination with others, may impact home range size (Aronsson et al., 2016; Molinari‐Jobin et al., 2007).

### 4.3 Scavengers

We found a significant effect of the presence of two of the large scavengers on foraging parameters. An overlap with areas where wild boars and brown bears occurred led to shorter handling times, which is most likely connected with kleptoparasitism. The presence of wild boar also shortened inter‐kill intervals. These results are in line with previous local studies focusing on the impact of kleptoparasitism by dominant scavengers on lynx at individual kills (Duľa & Krofel, 2020; Jędrzejewski et al., 1993; Krofel et al., 2012; Krofel & Jerina, 2016; Mattisson, Andren, et al., 2011), and other felids (e.g. mountain lion and leopard *Panthera pardus*; Allen et al., 2021; Smith et al., 2017; Tarugara et al., 2021). Since the capacity for lynx to compensate for losses from kleptoparasitism by increasing kill rates is limited (Krofel et al., 2012), scavenging could be especially relevant given recent rapid increases in the numbers of scavenger, such as the wild boar (Valente et al., 2020), across Europe. However, further research is needed to explore at what levels kleptoparasitism by any of the possible scavenger species start to translate into fitness costs for the lynx.

### 4.4 Human impact

We found a significant effect of human impact on the time lynx spends at a given kill site, with higher levels of pressure leading to shorter handling times, probably because lynx abandoned disturbed kill sites or, to a lesser extent, humans removed the prey remains. These results are consistent with previous observations from local studies on lynx (Belotti et al., 2018; Krofel et al., 2008; Tallian et al., 2023) and other felid species (e.g. mountain lions, Smith et al., 2015, 2017). However, the impact was not uniform across all populations. As expected, populations with small gradients of human impact did not show a high sensitivity to this parameter (e.g. Alpine), whereas populations with larger gradients of human impact (e.g. BBA and Carpathian) showed a higher sensitivity (Figure S4). This indicates the importance for including datasets from large environmental gradients to explore the effects of human activities on foraging parameters of predators. On the other hand, inter‐kill intervals were not affected by human impact, suggesting that people do not have a (consistent) influence on the searching time.

Overall, our results suggest that the main negative impact of human presence is related to predators' energy uptake, as shorter handling times are directly linked to reduced consumption of prey (Smith et al., 2015, 2017). This indicates that areas with high human pressure could be less optimal for lynx, not only due to increased mortality threats but also because of constraints imposed on their energy budgets.

### 4.5 Limitations

Despite the effort of generating a comparable dataset across populations, our analyses had limitations. Namely, although we obtained a relatively high accuracy in the predictive model to predict and classify ungulate kills, along with balanced sensitivity/specificity levels, some of the kill sites were not detected or were erroneously predicted as small prey during the cluster analysis (Table S2) likely due to short duration (e.g. ungulate kills abandoned early in the consumption process, for example, due to human disturbance or removal by scavengers; Oliveira et al., 2022). Thus, the effects of human disturbance and kleptoparasitism on lynx foraging parameters would likely have been stronger if such kills were considered. Additionally, we could not account for the different types of prey when classifying the clusters, which would have been relevant considering the prey diversity between and within study areas, since this parameter can affect foraging parameters.

We also faced limitations with standardising environmental layers across such a large scale. While we could obtain reliable and standardised layers at the European level regarding NDVI, human impact and habitat, it was not possible to obtain the same detailed and comparable information about the density of prey and scavengers across all studied populations. Therefore, we used proxies related to roe deer habitat preferences as a substitute for their abundance. However, there are other factors directly influencing roe deer (and other prey species) densities, such as seasonal spatial distribution, presence of other ungulate species and game management (Ferretti & Mori, 2020; Ossi et al., 2020). Despite the uncertainty associated to these layers, we believe that using three indexes for prey availability provided better results, and it was important to reflect the variation in prey type and availability across the study areas, particularly as the real prey densities are not available.

Similarly, the use of only coarse scale absence/presence data on large scavengers, as well as not taking in consideration potential seasonal variations on their distribution, may limit our insights into the impact of kleptoparasitism on lynx foraging, which has already been shown to depend on scavenger densities at local scales (Krofel & Jerina, 2016). Furthermore, we did not include data from other scavengers that might be important in some populations (e.g. large birds (Mattisson, Andren, et al., 2011), as well as stray dogs and grey wolves *Canis lupus* in the Balkan and Baltic populations, respectively; unpublished data). Provided more accurate datasets for these parameters, we would probably see clearer effects on foraging parameters. Therefore, establishing standardised monitoring methods for prey and scavenger densities would be of great importance for predation studies. Additionally, accounting for prey density would provide crucial information related to interactions with other environmental factors, such as human impact and scavengers' presence, providing a better understanding of confounding ecological effects and the identification of context‐dependent factors. However, we acknowledge the enormous logistical challenges associated with estimating ungulate and scavenger densities across larger scales.

## 5 CONCLUSIONS

Our approach enabled us to study Eurasian lynx predation patterns across Europe and to include wide environmental gradients, spanning from northern boreal regions, with some of the lowest human impact in Europe, to Mediterranean ecosystems and highly human‐dominated landscapes. We showed that the effects of multiple ecological factors on foraging parameters can be consistent for some populations but context‐dependent for others, indicating local adaptations by lynx. Understanding such variations is crucial to implement effective management measures on a population level, as a pattern observed in one area might not be necessarily transferable to other regions. Our results also demonstrate the ability of this solitary felid to adapt to a wide range of environmental conditions to maintain adequate food intake despite the constraints from humans, dominant scavengers and variable prey availability. At the same time, our study revealed the often‐neglected impact of humans on foraging abilities of large predators, which would not always be detected on the local scale.

## AUTHOR CONTRIBUTIONS

Teresa Oliveira and Miha Krofel conceived the ideas and designed the methodology. Teresa Oliveira, Jenny Mattisson, Kristina Vogt, John Linnell, John Odden, Elisa Belotti, Ludek Bufka, Rok Černe, Martin Duľa, Urša Fležar, Andrej Gonev, Micha Herdtfelder, Marco Heurich, Lan Hočevar, Tilen Hvala, Tomáš Iľko, Raido Kont, Petr Koubek, Jarmila Krojerová‐Prokešová, Jakub Kubala, Marko Kübarsepp, Josip Kusak, Miroslav Kutal, Beňadik Machciník, Peep Männil, Dime Melovski, Paolo Molinari, Aivars Ornicāns, Aleksandar Pavlov, Maruša Prostor, Vedran Slijepčević, Peter Smolko, Branislav Tam and Miha Krofel collected the data; Teresa Oliveira, Julian Oeser and Joseph Premier did the data curation, Teresa Oliveira analysed the data; Teresa Oliveira and Miha Krofel led the writing of the manuscript. All authors contributed critically to the drafts and gave final approval for publication.

## FUNDING INFORMATION

This study was financed by the European Commission (project LIFE Lynx LIFE16 NAT/SL/000634 and LIFE Luchs LIFE13 NAT/DE/000755); by the Research Council of Norway (Norges Forskningsråd (grants no. 251112 and 281092), NINA basic funding project no. 160022/F40), the Norwegian Directorate for Nature Management (Miljødirektoratet), the Nature Protection Division of the County Governor's Office for Innlandet, Viken, Vestfold and Telemark, & Trøndelag County; by the charity foundation from Liechtenstein, hunting inspectorate of the Canton of Bern, Stotzer‐Kästli‐Stiftung, Zigerli‐Hegi‐Stiftung, Haldimann‐Stiftung, Zürcher Tierschutz, Temperatio‐Stiftung, Karl Mayer Stiftung, Stiftung Ormella; by the Ministry of the Environment of the Slovak Republic. Data curation was supported by the Regina Bauer Stiftung. TO was supported by the Portuguese Foundation for Science and Technology (FCT, grant no. SFRH/BD/144110/2019). MKr was supported by the Slovenian Research and Innovation Agency (grants no. N1‐0163 and J1‐50013). JKu was supported by Bernd Thies Foundation, Plitvice Lakes NP and Public Institution ‘Priroda’. MKut and MD were supported by the Ministry of Environment of the Czech Republic (Project No. CZ.05.4.27/0.0/0.0/20_139/0012815), by the SaveGREEN project supported by Interreg Danube Transnational Programme (No. DTP3‐314‐2.3), the Dean's office of the Faculty of Forestry and Wood Technology, Mendel University in Brno and Training Forest Enterprise Masaryk Forest Křtiny. JP was supported by a grant from the Riepe Foundation.

## CONFLICT OF INTEREST STATEMENT

The authors declare no conflicts of interest.

## STATEMENT OF INCLUSION

Our study brings together authors from several countries, including a total of 31 different institutions, and it includes scientists based in the country where the study was carried out. Data collection was carried out by all institutions. Whenever adequate, we cited literature previously published by scientists from a given region. All authors were included early on with the research to make sure that the diverse sets of perspectives they represent were considered.

## Supporting information


**Appendix S1:** Summary of the data considered for the analyses.
**Appendix S2:** Predictive model for ungulate kill sites.
**Appendix S3:** Covariates included in the models.
**Appendix S4:** AIC values and additional results.

## Data Availability

Data available from the Dryad Digital Repository https://doi.org/10.5061/dryad.6djh9w1bs (Oliveira et al., 2024).
