# Supplementary material for: Ecological and intrinsic drivers of foraging parameters of Eurasian lynx at a continental scale
Source: J Anim Ecol. 2024 Nov 26;94(1):154–67. doi: 10.1111/1365-2656.14228 (PMC11730767; doi:10.1111/1365-2656.14228)
Supplement: Supplementary file 1 — Appendix S1: Summary of the data considered for the analyses. Appendix S2: Predictive model for ungulate kill sites. Appendix S3: Covariates included in the models. Appendix S4: AIC values and additional results. [file JANE-94-154-s001.docx]

**Appendix S1:** *Summary of the data considered for the analyses*

**Table S1.** Summary of the data used for the analyses per population, in terms of number of individuals, tracking sequences, and total number of days within the tracking sequences. The main prey species (from field-checked kill sites) are presented per population, along with the number of field-checked kill sites within tracking sequences that were considered for building the predictive model (see Appendix S2). BBA stands for Bohemian-Bavarian-Austrian.

| **Population** | **Individuals (m/f)** | **Number of tracking sequences** | **Number of days within the tracking sequences** | **Main prey species (%) [number of field-checked kills]** |
| --- | --- | --- | --- | --- |
| *Alpine* | 12 / 12 | 75 | 7,719 | Chamois (*Rupicapra rupicapra*; 41.8%), roe deer (*Capreolus capreolus*; 35.8%), lagomorphs^a^ (9.4%), Alpine marmot (*Marmota marmota*; 6.8%), others^b^ (5.9%) [n = 935] |
| *Black Forest* | 2 / 0 | 5 | 451 | Roe deer (86.3%), chamois (6.9%), others^c^ (6.9%) [n = 73] |
| *Balkan* | 7 / 5 | 29 | 3,345 | Roe deer (70.2%), brown hare (23.60%), others^d^ (6.2%) [n = 161] |
| *Baltic* | 14 / 3 | 48 | 3,201 | Roe deer (83.1%), mountain hare (7.7%), others^e^ (9.3%) [n = 443] |
| *BBA* | 4 / 3 | 15 | 408 | Roe deer (74.0%), red deer (*Cervus elaphus*, 16.0%), others^f^ (10.0%) [n = 50] |
| *Carpathian* | 14 / 2 | 41 | 4,925 | Roe deer (65.5%), red deer (22.4%), mouflon (6.7%), others^g^ (5.5%) [n = 255] |
| *Dinaric* | 16 / 5 | 38 | 3,532 | Roe deer (83.0%), red deer (7.1%), chamois (3.6%), others^h^ (6.4%) [n = 141] |
| *Central Scandinavia* | 5 / 1 | 14 | 477 | Roe deer (39.8%), semi-domestic reindeer (*Rangifer tarandus*; 27.3%), mountain hare (11.4%), moose (*Alces alces*; 5.7%), birds (3.4%), others^i^ (12.5%) [n = 88] |
| *South Scandinavia* | 12 / 5 | 43 | 1,321 | Domestic sheep (*Ovies aries*; 33.8%), roe deer (31.0%), mountain hare (23.2%), others^j^ (11.8%) [n = 142] |
| ***Overall*** | **86 / 36** | **308** | **25,379** | [n = 2288] |

(a) Brown hare *Lepus europaeus*, mountain hare *Lepus timidus*; (b) red fox *Vulpes vulpes*, domestic goat, birds, squirrel, other rodentia, unknown ungulates; (c) mouflon, unknown prey; (d) chamois, red fox, birds; (e) brown and unknown hare, beaver *Castor fiber*, capercaillie *Tetrao urogallus*, red fox, raccoon dog *Nyctereutes procyonoides*, birds; (f) brown hare, wild boar *Sus scrofa*; (g) unknown prey, unknown ungulate, wild boar, red fox, birds, fallow deer *Dama dama*; (h) red fox, unknown ungulates, mustelids, brown hare, domestic cat *Felis catus*; (i) capercaillie, black grouse (*Tetrao tetrix*), willow ptarmigan (*Lagopus lagopus*) other birds, red deer, red fox, domestic sheep other small mammals.

**
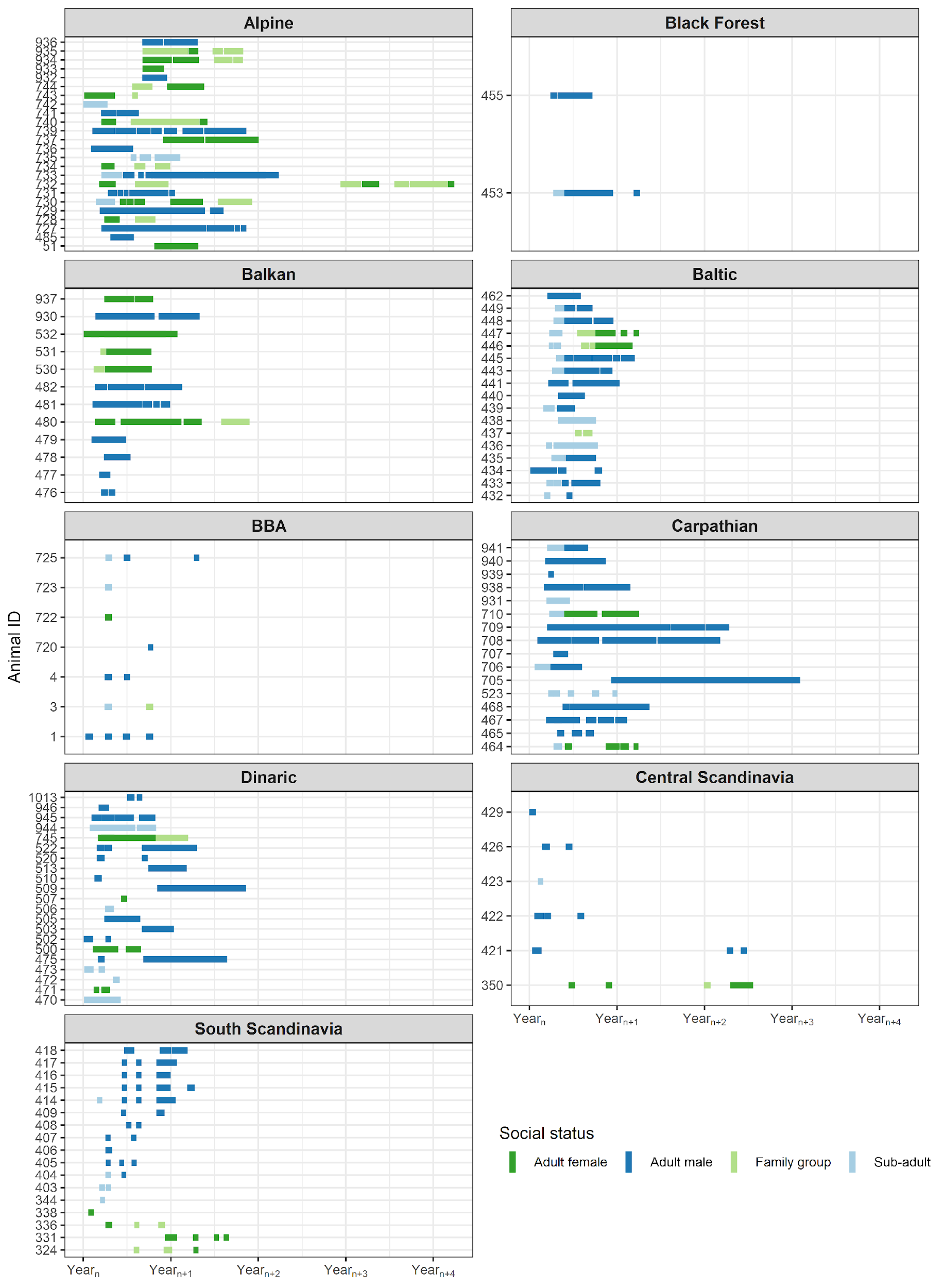
**

**Figure S1.** Tracking sequences (n=308), by population and social status (adult male, adult female, family group (mother with kittens), sub-adults - i.e., lynx less than two years old), obtained after the initial filtering of the data. Note that the status of the same animal could change during the deployment period.

*Animal captures and permit numbers*

Alpine population: Switzerland - Project north-western Alps III 2012-2014 - Lynx were captured following established standard protocols (described in Breitenmoser & Haller, 1993; Ryser et al., 2005; Ryser-Degiorgis et al., 2002; Zimmermann, Breitenmoser-Würsten & Breitenmoser, 2005) and with all permits required according to Swiss legislation for capturing, immobilizing, and radio tagging lynx (capture permits from the Federal Office for the Environment: Bewilligung_KORA_Luchsfang_BE_2010/2011/2006-03219/02/05/03, Bewilligung_KORA_Luchsfang_Kompartimente I, III und IV_2011-2015; animal experimentation permit from the Animal Welfare Commission ofthe Office for Agriculture and Nature of the Canton of Bern: 109/10 and 111/13). Lynx chamois project 2015-2018 - Lynx were captured following established standard protocols (described in Breitenmoser & Haller, 1993; Ryser et al., 2005; Ryser-Degiorgis et al., 2002; Zimmermann, Breitenmoser-Würsten & Breitenmoser, 2005) and with all permits required according to Swiss legislation for capturing, immobilizing, and radio tagging lynx (capture permits from the Federal Office for the Environment: Bewilligung_KORA_Luchsfang_Kompartimente I, III und IV_2011-2015, Bewilligung_KORA_Luchsfang_Schweiz_2017-2020/Q342-1667; animal experimentation permit from the Animal Welfare Commission of the Office for Agriculture and Nature of the Canton of Bern: 111/13 and BE3/17+). Italy - Italian Lynx Project - Lynx were captured following established standard protocols (described in Breitenmoser & Haller, 1993) and with all permits required according to Italian legislation for capturing, immobilizing, and radio tagging lynx (capture permits ISPRA 49392 and 53710).

Balkan population: The research programme in Macedonia was managed by the Macedonian Ecological Society. Trapping of the Balkan lynx was approved by the Macedonian Ministry of Environment and Physical Planning (permits number: 11-2186/2; 11-546/2;11-1006/10).

Baltic population: Latvia - The research program was managed by the Latvian State Forest Research Institute "Silava". Lynx captures were conducted in accordance with European and Latvian animal welfare laws. The study was designed to minimize animal stress and handling time, and to ensure animal welfare, as defined in the guidelines for the ethical use of animals in research. Permissions for lynx trapping were issued by headquarter of the State Forest Service (SFS) (permit No. 1-10/225 and No. 16-9/318). In addition, trapping process was approved by local authority of the SFS (No. 08-2/333). Estonia - The research program was managed by Estonian Environment Agency. Lynx captures were conducted in accordance with European and Estonian animal welfare laws. The study was designed to minimize animal stress and handling time, and to ensure animal welfare, as defined in the guidelines for the ethical use of animals in research. Permissions for lynx trapping were issued by Environmental Board (permit numbers: 1-4.1/13, 1-4.1/453, 1-4.1/12/540).

BBA population: The research program in the Bavarian Forest was managed by the Administration of the Bavarian Forest National Park. Lynx captures were conducted in accordance with European and German animal welfare laws. The experiment was designed to minimize animal stress and handling time, and to ensure animal welfare, as defined in the guidelines for the ethical use of animals in research. Animal captures and experimental procedures were approved by the Ethics Committee of the Government of Upper Bavaria and fulfils their ethical requirements for research on wild animals (Reference number 55.2-1-54-2531-82-10). The research program in the Bohemian Forest was managed by the Administration of the Sumava National Park. Lynx were captured following established standard protocols (described in: Belotti E, Weder N, Bufka L, Kaldhusdal A, Küchenhoff H, Seibold H, et al. (2015) doi:10.1371/journal.pone.0138139). The handling protocol was approved by the Czech Central Commission for Animal Welfare and fulfils their ethical requirements for research on wild animals (permit number: 55.2-1-54-2532-82-10). In addition, permits for wild animal capture were obtained from the Czech Central Commission for Animal Welfare (permit numbers: 44048/2008-17210, 44048/2008-10001) and the Czech Ministry of Environment (permit number: 41584/ENV/10-1643/620/10-PP8).

Black Forest: The research program in Baden-Wuerttemberg is managed by the Administration of the Forest Research Institute Baden-Wuerttemberg. Lynx captures are conducted in accordance with European and German animal welfare laws. The experiment was designed to minimize animal stress and handling time, and to ensure animal welfare, as defined in the guidelines for the ethical use of animals in research. Animal captures and experimental procedures were approved by the Ethics Committee of the governmental district of Freiburg and fulfils their ethical requirements for research on wild animals.

Carpathian population: The research program in the Moravian Karst was managed by the Mendel University in Brno. Lynx captures were conducted in accordance with European and Czech animal welfare laws. The experiment was designed to minimize animal stress and handling time, and to ensure animal welfare, as defined in the guidelines for the ethical use of animals in research. Permits for animal capture and handling were obtained from the PLA Moravian Karst Administration and the Ministry of Environment of the Czech Republic, permit numbers: SR/0081/JM/2017; 34128/ENV/17-2146/630/17. The research program in PLA Beskydy is managed by the Mendel University in Brno. Lynx captures were conducted in accordance with European and Czech animal welfare laws. The experiment was designed to minimize animal stress and handling time, and to ensure animal welfare, as defined in the guidelines for the ethical use of animals in research. Permits for animal capture and handling were obtained from the PLA Beskydy Administration and the Ministry of Environment of the Czech Republic, permit numbers: SR/0031/BE/2019; MZP/2020/630/167. The research program in PLA Beskydy was managed by the Institute of Vertebrate Biology of the Czech Academy of Sciences in Brno. Lynx captures were conducted in accordance with European and Czech animal welfare laws. The experiment was designed to minimize animal stress and handling time, and to ensure animal welfare, as defined in the guidelines for the ethical use of animals in research. Permissions for lynx captures were issued by the Protected Landscape Area Administration Beskydy (no. 6535/BE/2008) and approved by the Ethics Committee of the Czech Academy of Sciences (no. 130/2010).

Dinaric population: Slovenia - Lynx captures and translocations were conducted in accordance with European and Slovenian animal welfare laws. The study design aims to minimize animal stress and handling time, and to ensure animal welfare, as defined in the guidelines for the ethical use of animals in research. Animal captures and translocations, as well as experimental procedures were approved by the Slovenian Environmental Agency - Ministry of Environment and Spatial Planning, which issued permits No. 35601-29/2018-4 and 35601-90/2018-4. The research program in Slovenia is (also) managed by the University of Ljubljana. Lynx captures were conducted in accordance with European and Slovenian animal welfare laws. The experiment was designed to minimize animal stress and handling time, and to ensure animal welfare, as defined in the guidelines for the ethical use of animals in research. Animal captures and experimental procedures were approved by the committee at the Slovenian Environmental Agency, who also issued the research permits (no. 35601-45/2006-6 and 35601-76-2020-6). Croatia - The research program in Croatia is managed by the Faculty of Veterinary Medicine University of Zagreb. Lynx captures were conducted in accordance with European and Croatian animal welfare laws. The experiment was designed to minimize animal stress and handling time, and to ensure animal welfare, as defined in the guidelines for the ethical use of animals in research. Animal captures and experimental procedures were approved by the Ministry for Nature protection and the permit is renewed every two years.

Scandinavia population: The Norwegian part of the Scandinavian research project, SCANDLYNX, is managed by Norwegian Institute for Nature Research (NINA). All capture and handling procedures were approved by the Norwegian Experimental Animal Ethics Committee and followed their ethical requirements for research on wild animals (permit numbers (FOTS ID 2827, FOTS ID1391, 13912012/206992, 2010/161554, 2010/161563, 08/127430, 07/81885, 07/7883, 2004/48647, 201/01/641.5/FHB127/03/641.5/fhb, 1460/99/641.5/FBe, 1081/97/641.5/FBe, and NINA 1/95). In addition, permits to capture wild animals were provided by the Norwegian Environment Agency.

**Appendix S2:** *Predictive model for ungulate kill sites*

Following Oliveira et al. (2023), we built a model for predicting ungulate kill sites using the Random Forest algorithm and several cluster characteristics to build a binary predictive model able to distinguish between GLCs reflecting adult ungulate kills (1) from non-kills and small prey (0) (Oliveira et al., 2023; see Appendix S1-Table S1 for a list of prey species by population). We included most of the covariates considered in the previous study: cluster duration, fidelity to the cluster, maximum foray from the cluster’s centroid, average and maximum cluster radius, and proportion of night fixes (fixed – from 5 p.m. to 7 a.m. UTC points are classified as “night”, outside this period as “day”). Additionally, we included four other covariates: proportion of night fixes (automatic – according to the natural daylight), number of fixes within a cluster, latitude, and lynx status (four levels: adult male, adult female, family group - i.e., mother with kittens -, sub-adult - i.e., lynx less than two years old). We included the automatic proportion of night fixes because of the high variability of daylight at the same time across Europe, and because the predictive models performed better when including the two covariates related to the proportion of night fixes. The fixed proportion of night fixes was included to account for 24 hours of daylight in the most northern study areas in summer. We included the number of fixes within a cluster as this could influence our predictions (e.g. a kill site and a location where the animal passes frequently could have the same duration but different numbers of locations). We added latitude to account for potential differences between populations. Finally, we considered lynx status as this variable is more informative than simply considering lynx sex (two levels), and potentially important for cluster prediction. We split our dataset into 75%/25% similarly across populations to create train/test datasets to build the models and to evaluate model performance, respectively. We obtained a model accuracy of 85.53% (CI 95%: 82.98 – 87.83), with sensitivity and specificity of 84.56% and 86.54%, respectively. We show in Table S2 the confusion matrices for all populations and for each population separately. We show the importance of cluster attributes that explained the GLCs classification in Figure S2. All analyses and data visualisation were performed using R Statistical Software (v4.1.2; R Core Team 2021). We obtained the fixed night proportion from the *suncalc* v0.5.0 package (Thieurmel and Elmarhraoui, 2019). We used the GPSeqClus v1.3.0 package (Clapp et al., 2021) to generate the GLCs, randomForest v4.7-1.1 (Liaw and Wiener 2002) to build and evaluate the models, and randomForestExplainer v0.10.1 (Paluszynska et al., 2020) to obtain the importance of each variable.

**Table S2.** Confusion matrix for the test dataset for all populations and for each population separately. The GPS location cluster (GLC) has been classified correctly when the reference class of a GLC (field data) matches the class predicted for the same GLC by the model (diagonal). “0” reflects non-kills and small prey, and “1” reflects adult ungulates.

| All populations | | |  | | | |  | |  |  |
| --- | --- | --- | --- | --- | --- | --- | --- | --- | --- | --- |
|  | *Reference* | |  | |  |  |  | |  |  |
| *Prediction* | *0* | *1* |  | |  |  |  | |  |  |
| *0* | 367 | 56 |  | |  |  |  | |  |  |
| *1* | 67 | 360 |  | |  |  |  | |  |  |
| **1 - Alpine** |  |  | **2 -Balkan** | |  |  | **3 - Baltic** | |  |  |
|  | *Reference* | |  | *Reference* | | |  | *Reference* | | |
| *Prediction* | *0* | *1* | *Prediction* | | *0* | *1* | *Prediction* | | *0* | *1* |
| *0* | 208 | 27 | *0* | | 8 | 1 | *0* | | 8 | 7 |
| *1* | 38 | 139 | *1* | | 4 | 23 | *1* | | 13 | 76 |
| **4 - BBA** | | | **5 – Carpathian** | | | | **6 – Dinaric** | | | |
|  | *Reference* | |  | | *Reference* |  |  | | *Reference* |  |
| *Prediction* | *0* | *1* | *Prediction* | | *0* | *1* | *Prediction* | | *0* | *1* |
| *0* | 1 | 1 | *0* | | 6 | 1 | *0* | | 0 | 6 |
| *1* | 0 | 9 | *1* | | 4 | 53 | *1* | | 3 | 26 |
| **7 - Black Forest** | | | **8 – South Scandinavia** | | | | **9 – Central Scandinavia** | | | |
|  | *Reference* | |  | | *Reference* | |  | | *Reference* |  |
| *Prediction* | *0* | *1* | *Prediction* | | *0* | *1* | *Prediction* | | *0* | *1* |
| *0* | 0 | 2 | *0* | | 84 | 6 | *0* | | 52 | 5 |
| *1* | 0 | 11 | *1* | | 2 | 14 | *1* | | 3 | 9 |


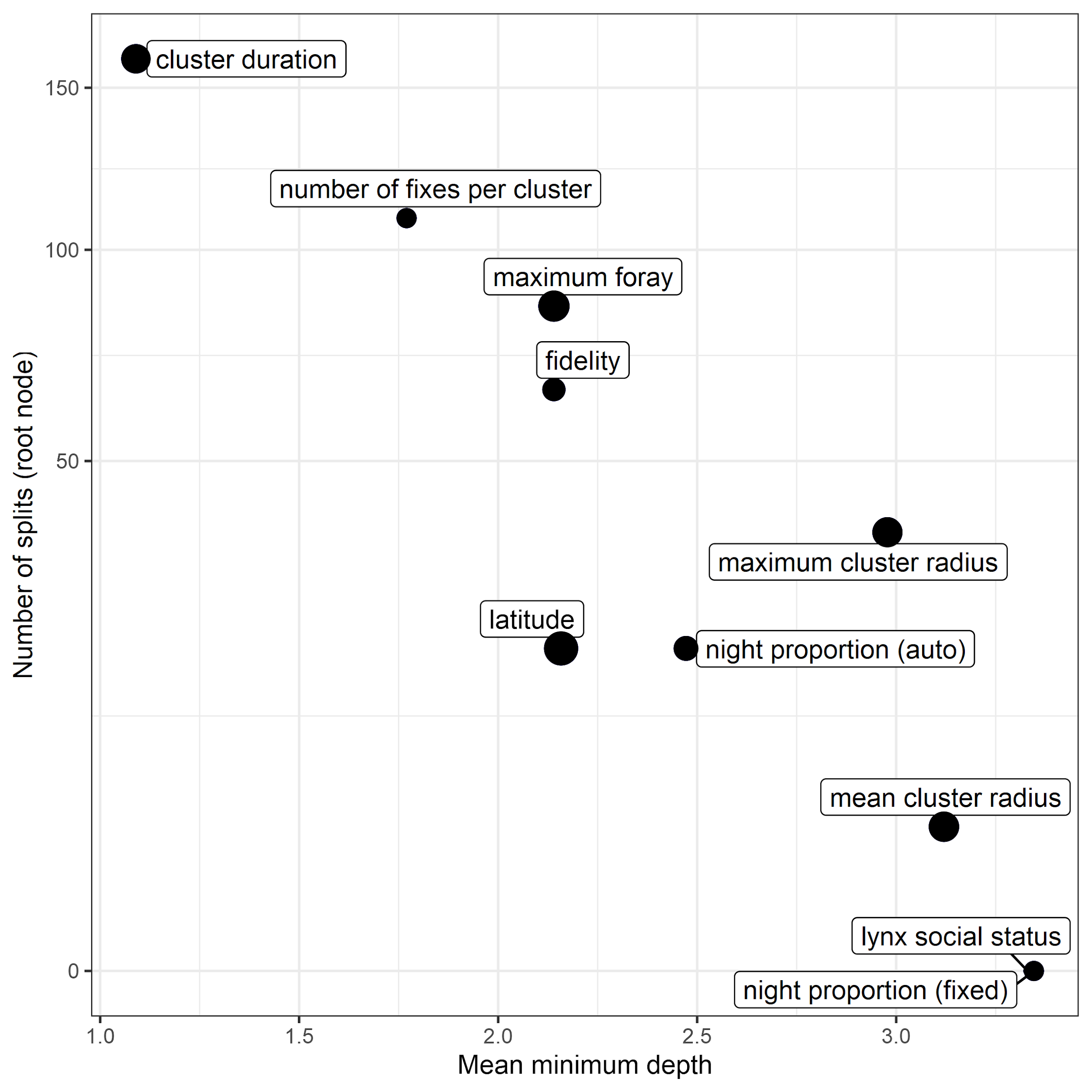


**Figure S2.** Multi-way importance plot for the predictive model. The x-axis shows the mean minimal depth of the first split on a given variable and the y-axis shows the number of times a variable is used to split the root node. The circle’s area is proportional to the number of nodes that use a variable for splitting. Higher values on y, lower values on x, and larger symbols indicate higher importance of the cluster attribute for classification. Thus, variables in the top left corner are more important for GLCs classification.

**References**

Clapp, G. J (2022). _GPSeqClus: Sequential Clustering Algorithm for Location Data_. R package version 1.3.0, <https://CRAN.R-project.org/package=GPSeqClus>.

Liaw, A. & Wiener, M. (2002). Classification and Regression by randomForest. R News 2(3), 18-22.

Oliveira, T., Carricondo‐Sanchez, D., Mattisson, J., Vogt, K., Corradini, A., Linnell, J. D., ... & Krofel, M. (2023). Predicting kill sites of an apex predator from GPS data in different multiprey systems. Ecological Applications, 33(2), e2778.

Paluszynska A., Biecek P., & Jiang Y. (2020). _randomForestExplainer: Explaining and Visualizing Random Forests in Terms of Variable Importance_. R package version 0.10.1, <https://CRAN.R-project.org/package=randomForestExplainer>.

Thieurmel, B., and A. Elmarhraoui. 2019. “suncal: Compute Sun Position, Sunlight Phases, Moon Position and Lunar Phase.” R Package, v0.5.0. https://cran.r-project.org/web/packages/ suncalc/suncalc.pdf.

**Appendix S3:** *Covariates included in the models*

**Table S3.** Description and units of each covariate included in the models and the parameters for which a given covariate was extracted and tested. For each tracking sequence, we created a 95% MCP of the GPS data within the tracking sequence. The abbreviation “IK” stands for inter-kill interval, while “HT” stands for handling time.

| Variable | Proxy for | Parameters | Description | Type/Units [Source] |
| --- | --- | --- | --- | --- |
| Social status | - | IK, HT | Social status of each lynx in a given tracking sequence | Categorical (adult male, adult female, family group) [data-driven] |
| Population id | - | IK, HT | Population where the lynx is included | Categorical [data-driven] |
| Month | - | IK, HT | Month extracted from the date of each cluster (first GPS fix) | Continuous / January to December [data-driven] |
| Home-range area (HR) | prey availability | IK | 85% MCP of all GPS data for an individual with more than 10 months of tracking data. When an individual did not fit in this criterion, we attributed, as its home range size, the average HR size within the population and sex. | Continuous / km^2^ [data-driven] |
| Normalized Difference Vegetation Index (NDVI) | prey availability | IK | NDVI values (annual mean) extracted across each tracking sequence (95% MCP) | Continuous / 250x250m [Didan, 2021] |
| Forest edge density (FED) | prey availability | IK | Forest edge density calculated for each tracking sequence (95% MCP; median value) | Continuous / m/km^2^ [estimated following Ruiz-Villar et al., 2023] |
| Human modification Index (HMI) | human disturbance | IK, HT | Human modification index extracted across each tracking sequence (95% MCP; median value) and for each kill | Continuous, 300x300m [Theobald et al., 2020] |
| Distance to settlements (DIST_S) | human disturbance | IK, HT | Distance to settlements (includes classes for built-up areas, Ferri et al., 2017) calculated across each tracking sequence (95% MCP; median value) and for each kill | Continuous, m [Ferri et al., 2017] |
|  |  |  |  |  |
| Bear proportion | scavengers presence | IK, HT | Proportion of the area of each tracking sequence (95% MCP) overlapping with bear occurrence map | Continuous (%) [Kaczensky et al., 2021, presence/absence source layer with 10x10 km] |
| Wild boar proportion | scavengers presence | IK, HT | Proportion of the area of each tracking sequence (95% MCP) overlapping with wild boar occurrence map | Continuous (%) [Linnell et al., 2020, presence/absence source layer with 10x10 km] |
| Wolverine proportion | scavengers presence | IK, HT | Proportion of the area of each tracking sequence (95% MCP) overlapping with wolverine occurrence map | Continuous (%) [Kaczensky et al., 2021, presence/absence source layer with 10x10 km] |
| Scavengers count | scavengers presence | HT | Number of scavenger species (bear, wild boar, wolverine) where occurrence map overlaps the location of the kill site | Categorical (0 – no scavengers, 1 – one scavenger species, 2 – two scavenger species in the area) [Linnell et al., 2020, Kaczensky et al., 2021] |

**References**

Ferri, S., Siragusa, A., Sabo, F., Pafi, M., & Halkia, S. (2017). The European Settlement Map 2017 Release; Methodology and output of the European Settlement Map (ESM2p5m). doi:10.2760/780799

Linnell, J. D., Cretois, B., Nilsen, E. B., Rolandsen, C. M., Solberg, E. J., Veiberg, V., ... & Kaltenborn, B. (2020). The challenges and opportunities of coexisting with wild ungulates in the human-dominated landscapes of Europe's Anthropocene. Biological Conservation, 244, 108500.

Grantham, H. S., Duncan, A., Evans, T. D., Jones, K. R., Beyer, H. L., Schuster, R., ... & Watson, J. E. M. (2020). Anthropogenic modification of forests means only 40% of remaining forests have high ecosystem integrity. Nature communications, 11(1), 5978.

Kaczensky, Petra et al. (2021). Distribution of large carnivores in Europe 2012 - 2016: Distribution maps for Brown bear, Eurasian lynx, Grey wolf, and Wolverine, Dryad, Dataset, https://doi.org/10.5061/dryad.pc866t1p3

Ruiz-Villar, H., Bastianelli, M. L., Heurich, M., Anile, S., Díaz-Ruiz, F., Ferreras, P., ... & Palomares, F. (2023). Agriculture intensity and landscape configuration influence the spatial use of wildcats across Europe. Biological Conservation, 277, 109854.

Theobald, D. M., Kennedy, C., Chen, B., Oakleaf, J., Baruch-Mordo, S., & Kiesecker, J. (2020). Earth transformed: detailed mapping of global human modification from 1990 to 2017. *Earth System Science Data*, *12*(3), 1953-1972


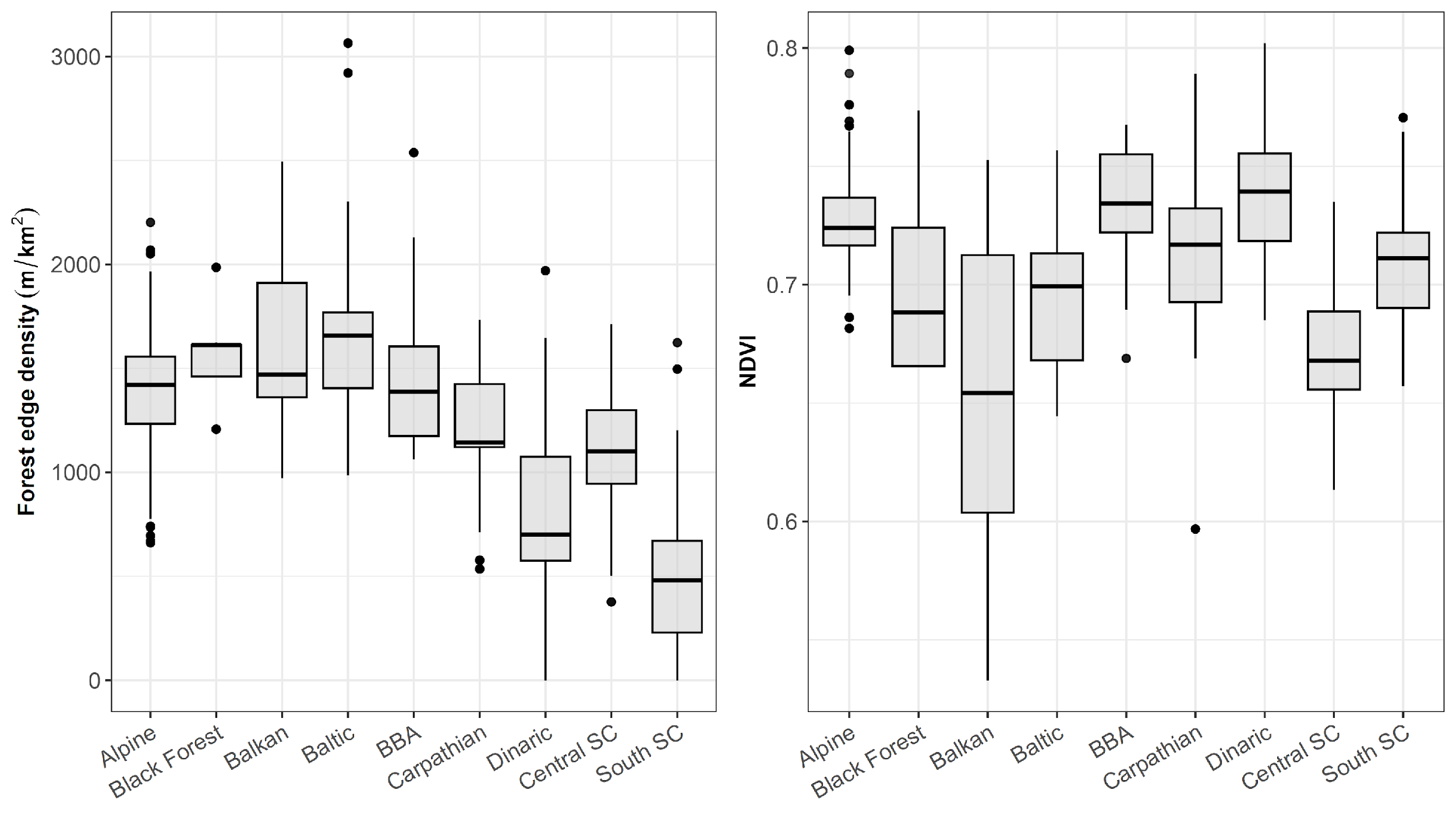


**Figure S3.** Boxplots showing the median availability of forest edge density (left panel) and NDVI (right panel) across populations, within tracking sequences.


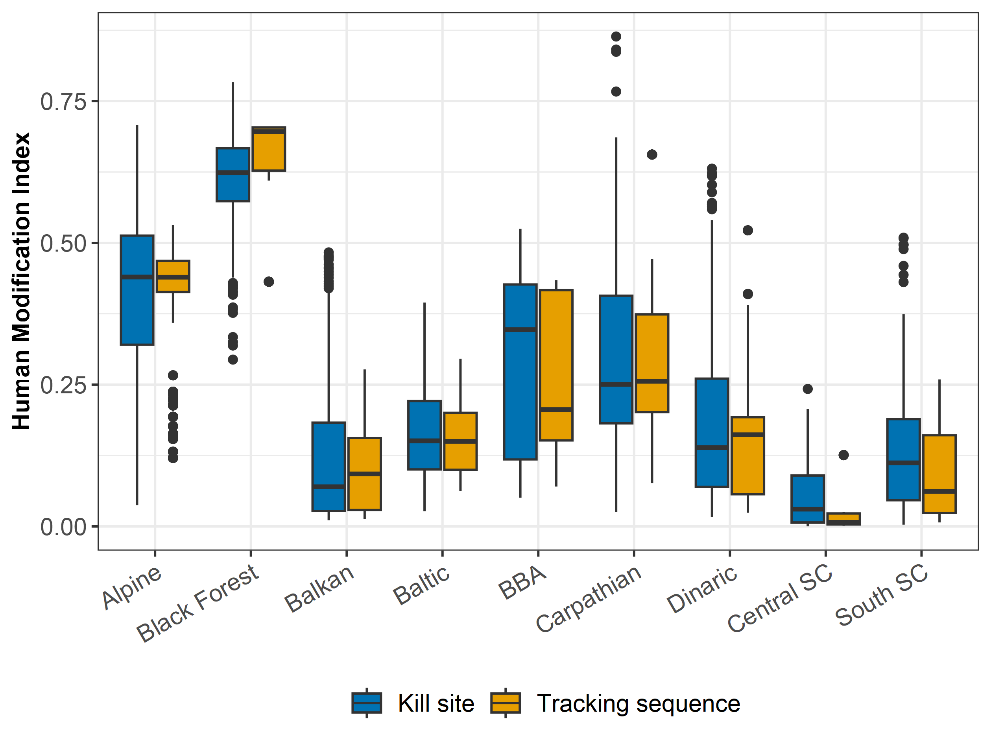


**Figure S4.** Boxplot showing the human modification index values across populations, by kill sites and by tracking sequences (median value).


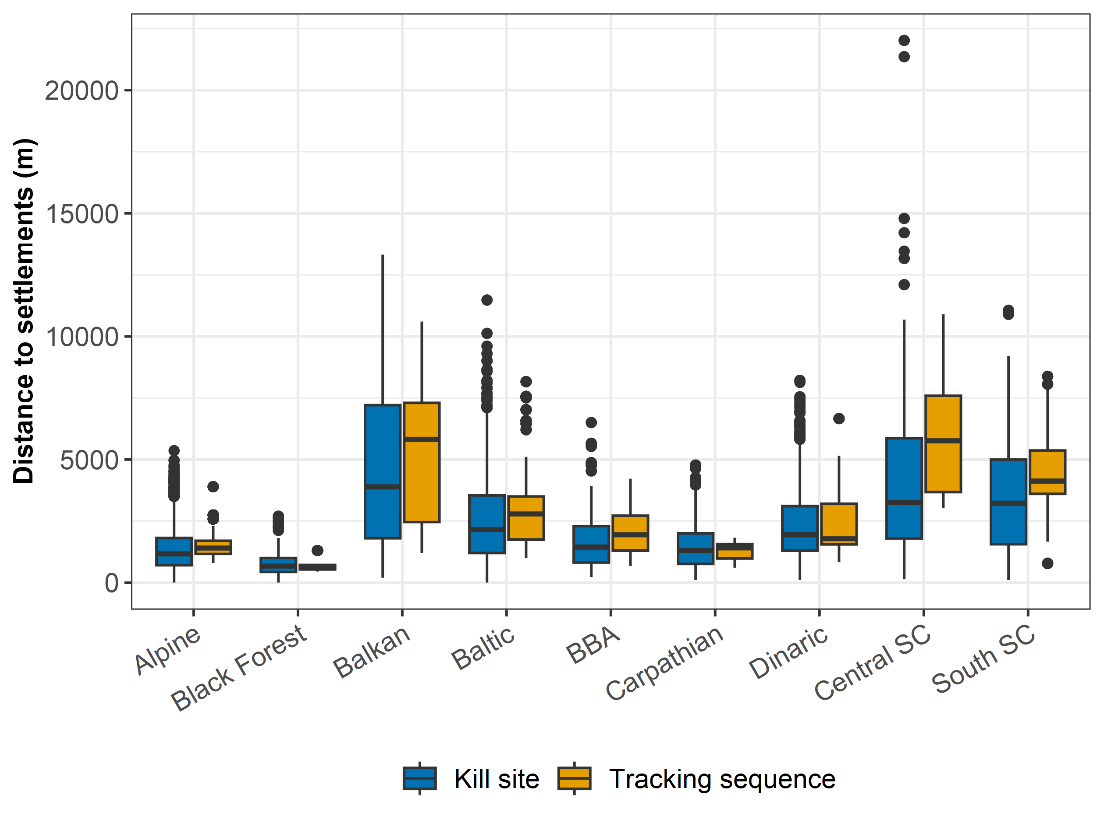


**Figure S5.** Boxplot showing the distance to settlements across populations, by kill sites and within tracking sequences (median value).


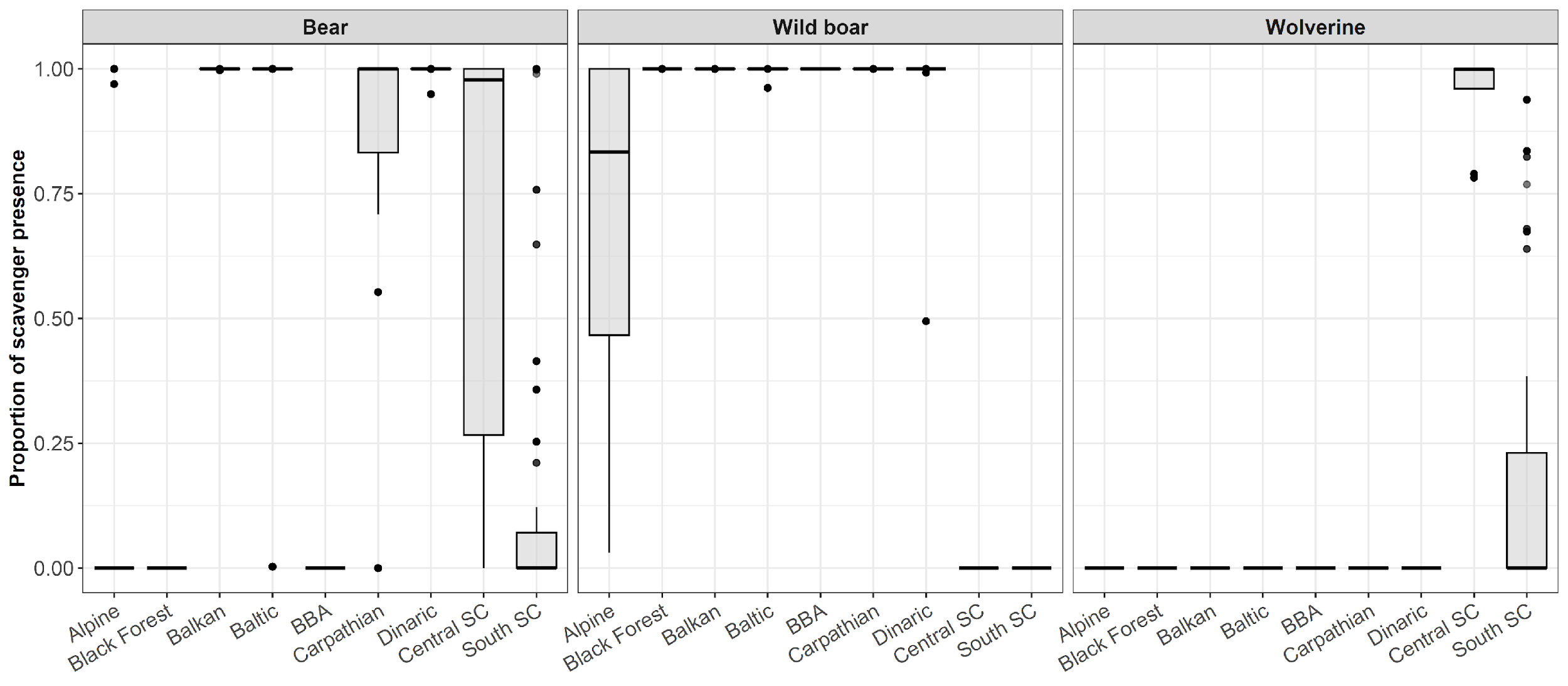


**Figure S6.** Boxplots with the proportion of scavenger presence across populations, within tracking sequences.

**Appendix S4:** *AIC values and additional results*

**
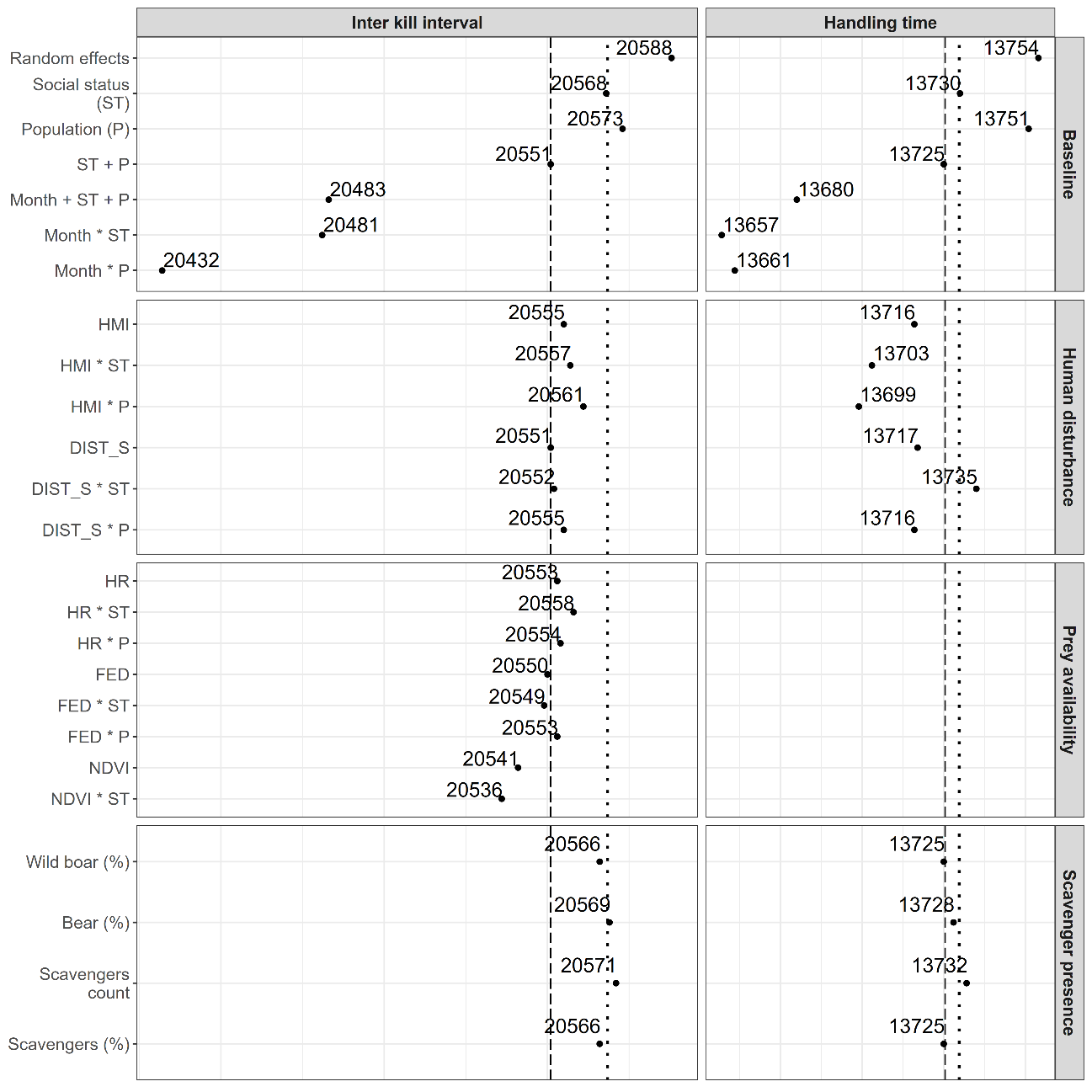
**

**Figure S7.** Comparison of model support (AIC values) for different model setups, for inter-kill interval and handling time. The random effects include animal id and number of fixes per day when considering the inter-kill interval as a response variable, while for handling time we included only animal id (as the number of fixes did not improve the model). The dashed line marks the AIC value for the model with social status (ST) and population ID (P) only, to which the models within prey availability and human disturbance should be compared (both include parametric effects). The dotted line marks the AIC value for the model including only social status, to which the models within the scavengers’ presence should be compared to, as population ID was not included in the scavengers’ models (due to correlation). “HR” stands for home range (85% MCP), “HMI” stands for human modification index, “DIST_S” for distance to settlements, “FED” for forest edge density, and “NDVI” for normalized difference vegetation index. The scavengers’ models were built without the smooth factor for that covariate (edf~1). Models with “*” indicate the factor-smooth interaction with either social status or population. We did not include a model with a factor-smooth interaction between NDVI and population id because we did not have a good representation for the lower NDVI values for all populations (Fig. S3). We did not consider wolverine as a single species covariate since only a few individual lynx in our dataset overlapped with wolverines in their ranges (limited to parts of Central and South Scandinavia, n = 23).

**Table S4.** GAMs formulas for the covariates (Social status, population, month, human modification index (HMI), distance to settlements (DIST_S), home range size (HR), forest edge density (FED), normalized difference vegetation index (NDVI), bear and wild boar proportion within the tracking sequence, and scavengers count). “s” stands for the inclusion of a smooth term where bs indicates the basis type used (“cc” - “cyclic spines”, “re” - “random effects “, “fs” - “factor smooth “) and k stands for the number of basis functions to be computed. The random effects included animal id (“id), and the number of fixes (“n_fix”).

|  | **Covariates** | **Covariates model formula** |
| --- | --- | --- |
| **Baseline** | Random effects | s(id, bs=”re”) + s(n_fix, bs="re") |
|  | Social Status (ST) | ST + s(id, bs=”re”) + s(n_fix, bs="re") |
|  | Population (P) | P + s(id, bs=”re”) + s(n_fix, bs="re") |
|  | ST + P | ST + P + s(id, bs=”re”) + s(n_fix, bs="re") |
|  | Month + ST + P | ST + P + s(Month, bs="cc", k = 12) + s(id, bs=”re”) + s(n_fix, bs="re") |
|  | Month * ST | s(Month, bs="cc", k = 12) + P + s(Month, k = 12, ST, bs="fs") + s(id, bs=”re”) + s(n_fix, bs="re") |
|  | Month * P | s(Month, bs="cc", k = 12) + ST + s(Month, k = 12, P, bs="fs") + s(id, bs=”re”) + s(n_fix, bs="re") |
| **Human disturbance** | HMI | s(HMI) + ST + P + s(id, bs=”re”) + s(n_fix, bs="re") |
|  | HMI * ST | s(HMI) + P + s(HMI, ST, bs="fs") + s(id, bs=”re”) + s(n_fix, bs="re") |
|  | HMI * P | s(HMI) + ST + s(HMI, P, bs="fs") + s(id, bs=”re”) + s(n_fix, bs="re") |
|  | DIST_S | s(DIST_S) + ST + P + s(id, bs=”re”) + s(n_fix, bs="re") |
|  | DIST_S * ST | s(DIST_S) + P + s(DIST_S, ST, bs="fs") + s(id, bs=”re”) + s(n_fix, bs="re") |
|  | DIST_S * P | s(DIST_S) + ST + s(DIST_S, P, bs="fs") + s(id, bs=”re”) + s(n_fix, bs="re") |
| **Prey availability** | HR | s(HR) + ST + P + s(id, bs=”re”) + s(n_fix, bs="re") |
|  | HR * ST | s(HR) + P + s(HR, ST, bs="fs") + s(id, bs=”re”) + s(n_fix, bs="re") |
|  | HR * P | s(HR) + ST + s(HR, P, bs="fs") + s(id, bs=”re”) + s(n_fix, bs="re") |
|  | FED | s(FED) + ST + P + s(id, bs=”re”) + s(n_fix, bs="re") |
|  | FED * ST | s(FED) + P + s(FED, ST, bs="fs") + s(id, bs=”re”) + s(n_fix, bs="re") |
|  | FED * P | s(FED) + ST + s(FED, P, bs="fs") + s(id, bs=”re”) + s(n_fix, bs="re") |
|  | NDVI | s(NDVI) + ST + P + s(id, bs=”re”) + s(n_fix, bs="re") |
|  | NDVI * ST | s(NDVI) + P + s(NDVI, ST, bs="fs") + s(id, bs=”re”) + s(n_fix, bs="re") |
|  | NDVI * P | s(NDVI) + ST + s(NDVI, P, bs="fs") + s(id, bs=”re”) + s(n_fix, bs="re") |
| **Scavenger presence** | Wild boar (%) | Wild_boar + ST + s(id, bs=”re”) |
|  | Bear (%) | Bear + ST + s(id, bs=”re”) |
|  | Scavenger count | Scavenger_count + ST + s(id, bs=”re”) |
|  | Scavengers (%) | Wild_boar + Bear + ST + s(id, bs=”re”) |


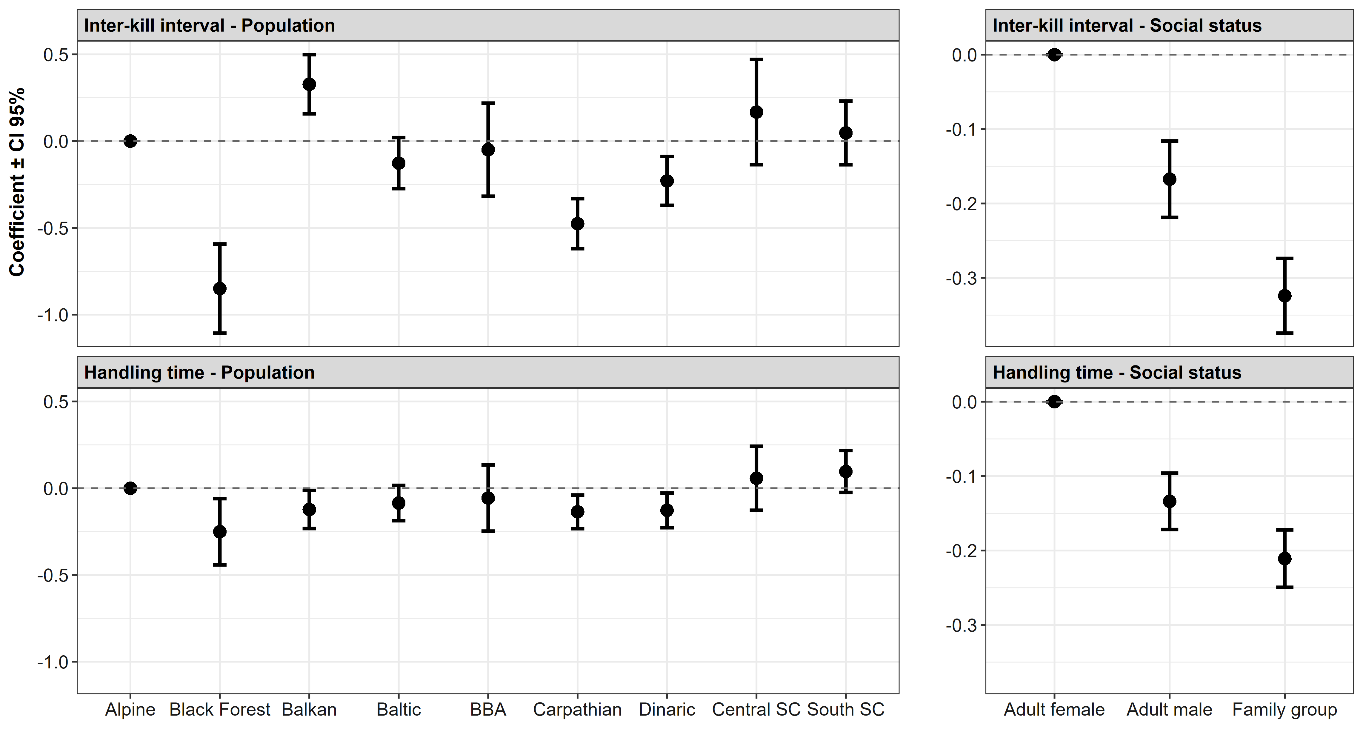


**Figure S8.** Estimated coefficients (± CI 95%) for the parametric coefficients of the model including population ID and social status (Figure S7). BBA stands for Bohemian-Bavarian-Austrian population. The Alpine population and single adult female are the reference categories, and confidence intervals crossing zero reflect non-significant differences when compared to the reference category. Note that data from the Black Forest population is limited to two males.


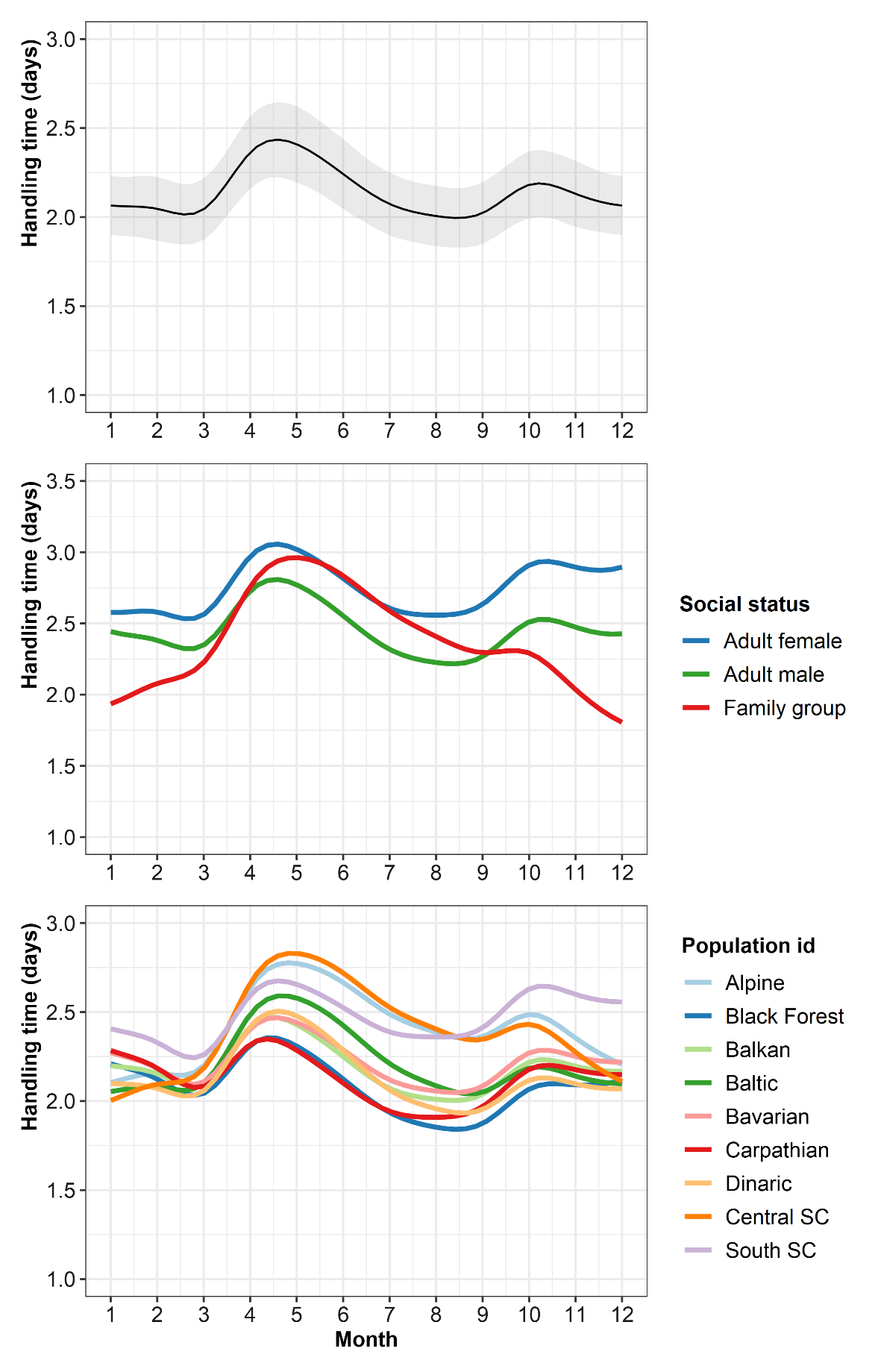


**Figure S9.** Variation of the predicted values for handling time along the year (first panel), by social status (second panel), and by population (third panel). The reference category for social status is adult male (first and third panel) and for population id (first and second panel) is the Carpathian population. Note that data from the Black Forest population is limited to two males. Note that family groups do not include data during the denning period (two months, starting around May). Therefore, data during these months is predicted from the other available data.


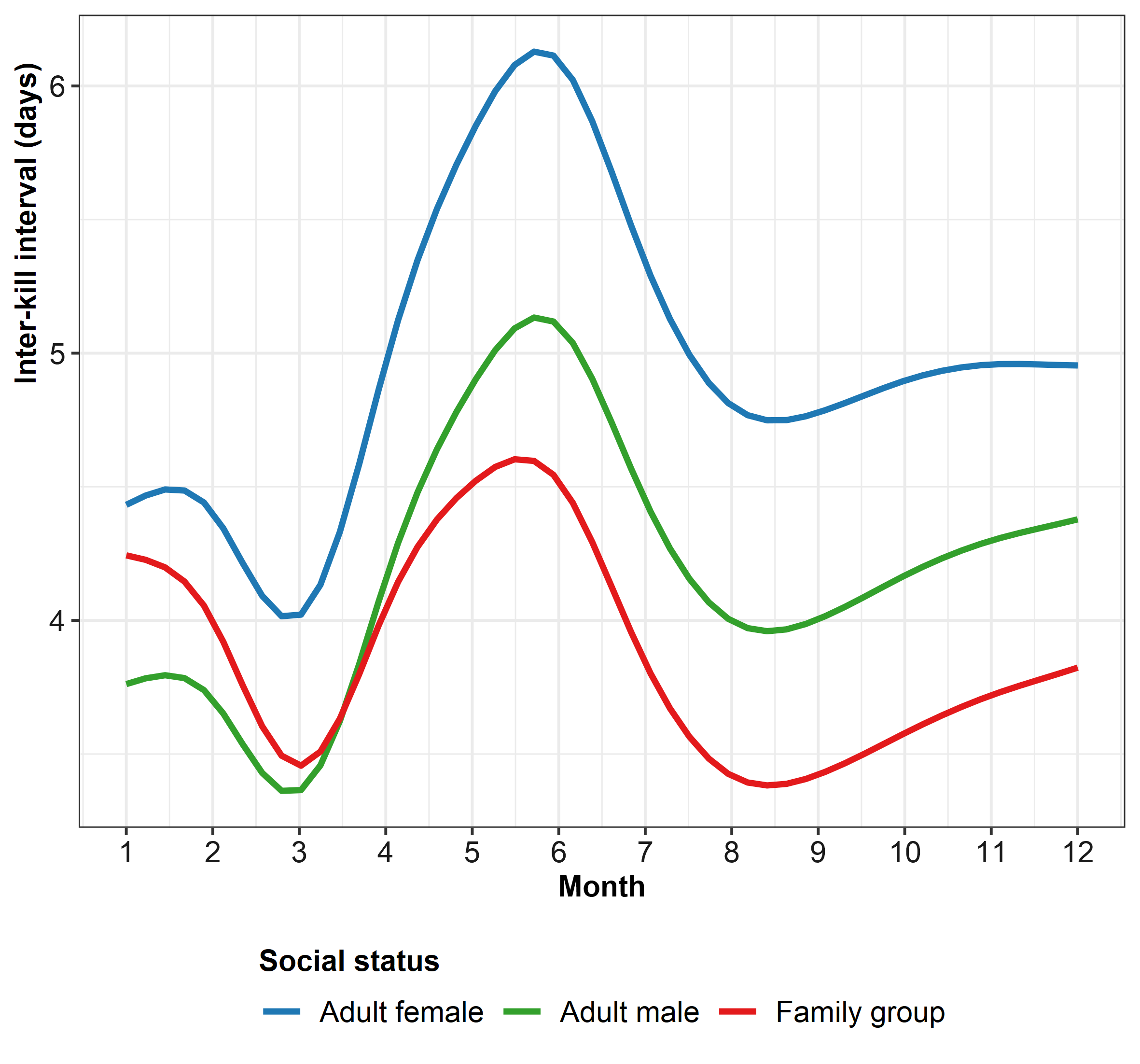


**Figure S10.** Variation of the predicted values for inter-kill interval by social status. Longer inter-kill intervals reflect lower kill rates. The reference category for population ID is the Carpathian population. Note that family groups do not include data during the denning period (two months, starting around May). Therefore, data during these months is predicted from the other available data.


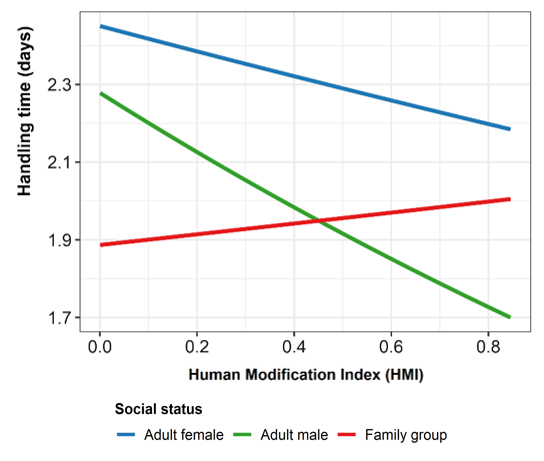


**Figure S11.** Variation of the predicted values for handling time with human modification index (HMI) by social status. The reference category for population id is the Carpathian population.
